# Calcium-induced differentiation in normal human colonoid cultures: Cell-cell / cell-matrix adhesion, barrier formation and tissue integrity

**DOI:** 10.1371/journal.pone.0215122

**Published:** 2019-04-17

**Authors:** Durga Attili, Shannon D. McClintock, Areeba H. Rizvi, Shailja Pandya, Humza Rehman, Daniyal M. Nadeem, Aliah Richter, Dafydd Thomas, Michael K. Dame, Danielle Kim Turgeon, James Varani, Muhammad N. Aslam

**Affiliations:** 1 Department of Pathology, The University of Michigan Medical School, Ann Arbor, Michigan, United States of America; 2 Department of Internal Medicine, The University of Michigan Medical School, Ann Arbor, Michigan, United States of America; Università degli Studi della Campania, ITALY

## Abstract

**Background and aims:**

The goal of the study was to assess calcium alone and Aquamin, a multi-mineral natural product that contains magnesium and detectable levels of 72 trace elements in addition to calcium, for capacity to affect growth and differentiation in colonoid cultures derived from histologically-normal human colon tissue.

**Methods:**

Colonoid cultures were maintained in a low-calcium (0.25 mM) medium or in medium supplemented with an amount of calcium (1.5–3.0 mM), either from calcium alone or Aquamin for a period of two weeks. This was shown in a previous study to induce differentiation in colonoids derived from large adenomas. Changes in growth, morphological features and protein expression profile were assessed at the end of the incubation period using a combination of phase-contrast and scanning electron microscopy, histology and immunohistology, proteomic assessment and transmission electron microscopy.

**Results:**

Unlike the previously-studied tumor-derived colonoids (which remained un-differentiated in the absence of calcium-supplementation), normal tissue colonoids underwent differentiation as indicated by gross and microscopic appearance, a low proliferative index and high-level expression of cytokeratin 20 in the absence of intervention (i.e., in control condition). Only modest additional changes were seen in these parameters with either calcium alone or Aquamin (providing up to 3.0 mM calcium). In spite of this, proteomic analysis and immunohistochemistry revealed that both interventions induced strong up-regulation of proteins that promote cell-cell and cell-matrix adhesive functions, barrier formation and tissue integrity. Transmission electron microscopy revealed an increase in desmosomes in response to intervention.

**Conclusions:**

These findings demonstrate that colonoids derived from histologically normal human tissue can undergo differentiation in the presence of a low ambient calcium concentration. However, higher calcium levels induce elaboration of proteins that promote cell-cell and cell-matrix adhesion. These changes could lead to improved barrier function and improved colon tissue health.

## Introduction

Epidemiological studies have demonstrated that calcium intake and colon polyp formation / colon cancer are inversely related [1–7]. In spite of this, interventional trials using calcium supplementation to reduce polyp formation have been only modestly effective. Some chemoprevention trials have shown a reduction in polyp incidence [8,9] but others have failed to find significant benefit [10,11]. One chemoprevention study actually found an increased risk of developing colon polyps with the sessile-serrated phenotype—a pathological presentation associated with increased risk of colon cancer—with calcium supplementation [12]. The same interventional trials have also demonstrated only modest (though in some cases, statistically significant) effect on biomarkers of growth and differentiation in histologically-normal colon tissue [13,14].

Why intervention with calcium is not more effective against colon polyp formation is not fully understood. One thought is that effective chemoprevention requires that there be an adequate level of dietary calcium throughout life. Once early molecular damage has occurred, it is too late to block polyp formation and progression to invasive cancer. Alternatively, calcium supplementation may lack better efficacy because optimal chemoprevention requires additional nutrients along with calcium. Vitamin D, for example, is well-known to be required for calcium uptake and utilization. A vitamin D deficiency is associated with many of the same maladies as a calcium-deficiency, including colon cancer [15–18]. Other studies have demonstrated that certain trace elements nutritionally associated with calcium may contribute to polyp prevention. For example, the ratio of magnesium to calcium has been shown to be as important as the level of calcium itself for effective colon cancer chemoprevention in mice [19]. Other studies have demonstrated anti-tumor activity with copper, manganese and selenium [20,21]. Our own past studies have shown that members of the lanthanide family of “rare earth” elements enhance the growth-regulating activity of calcium for colon epithelial cells [22,23]. These, and other potentially important nutrients, would not be provided in a calcium supplement alone.

As part of our effort to understand the role of trace elements in colon polyp chemoprevention, we have conducted both *in vitro* and *in vivo* studies using Aquamin—a calcium-rich, multi-mineral product derived from red marine algae of the *Lithothamnion* family—as an intervention. Aquamin contains calcium and magnesium along with detectable levels of 72 additional trace elements [24]. In cell culture studies [25,26] better colon epithelial cell growth-regulating activity was seen and in animal studies [27,28] better polyp prevention activity was observed with Aquamin than with calcium alone.

Whether the combination of calcium and additional trace elements (be it Aquamin itself or some other mix of appropriate minerals) will, ultimately, prove to be more effective than calcium alone as a colon polyp chemopreventive agent in humans remains to be seen. A problem with translating preclinical findings to results in humans is the low incidence of colon polyp formation and the long lag period between initial molecular changes and outgrowth of observable lesions. Additionally, progression from initial polyp formation to more serious disease is difficult to study experimentally since colonic polyps are removed upon detection. Colonoid culture technology, which is now well-developed [29–32], provides a way to study human colon polyp responses to potentially useful chemopreventive agents under *ex vivo* conditions. In a recent study using human colon adenomas in colonoid culture, we found that supplementation of the culture medium with either calcium alone or Aquamin induced differentiation in the adenomas as indicated by a change in morphology and by increased expression of differentiation-related proteins [33]. With Aquamin, features of differentiation were observed at a lower concentration than was required with the equivalent level of calcium alone. With both interventions, differentiation was associated with reduced proliferation (as indicated by Ki67 staining) and with alterations in several growth-regulating pathways. Whether the differentiation-enhancing and growth-suppressing activity of Aquamin is unique to the abnormal epithelium or whether similar changes might also be seen in the normal colonic mucosa is not known. As a way to begin addressing this question, we have in the present study compared the effects of Aquamin to calcium alone for the ability to modulate growth and differentiation in colonoid cultures derived from specimens of histologically-normal human colonic mucosa. Understanding how colonoids derived from histologically-normal tissue respond to intervention is critical, ultimately, for understanding the significance of similar responses observed in the premalignant adenoma tissue.

## Materials and methods

### Aquamin

Aquamin is a calcium-rich, magnesium-rich multi-mineral product obtained from the skeletal remains of the red marine algae, *Lithothamnion sp* [24] (Marigot Ltd, Cork, Ireland). Aquamin contains calcium and magnesium in a ratio of approximately (12:1), along with measurable levels of 72 other trace minerals (essentially all of the minerals algae fronds accumulate from the deep ocean water). Mineral composition was established via an independent laboratory (Advanced Laboratories; Salt Lake City, Utah) using Inductively Coupled Plasma Optical Emission Spectrometry (*ICP***-***OES*). Our recently published study with adenoma colonoids provides a complete list of elements detected in Aquamin and their relative amounts [33], the same product used here to employ a multi-mineral approach. Aquamin is sold as a dietary supplement (GRAS 000028) and is used in various products for human consumption in Europe, Asia, Australia, and North America. A single batch of Aquamin Soluble was used for this study. Calcium Chloride 0.5 M solution (PromoCell GmbH, Heidelberg, Germany) was used as a source of calcium.

### Tissue samples for colonoid culture

Histologically normal colon tissue (2.5-mm biopsies) was taken from the sigmoid colon of five subjects by flexible sigmoidoscopy without prior colon preparation. The five subjects were from a group defined as at “increased risk for colon cancer based on a personal history of colonic polyps or colon cancer or a family (first degree blood relative) history of colon cancer”. The study was approved by the Institutional Review Board (IRBMED) at the University of Michigan (IRB# HUM00076276), and conducted according to the principles stated in the Declaration of Helsinki. All subjects provided written informed consent prior to biopsy. These subjects were also screened for use of calcium or vitamin D supplements, and any supplement use was stopped two weeks prior to biopsy. S1 Table provides demographic information.

### Establishment of colonoids from normal human colonic mucosa

Colonoid cultures were established from histologically-normal colon tissue based on our recently described methods [32–34]. Briefly, biopsies were finely minced on ice using a #21 scalpel and seeded into Matrigel (Corning), prepared to 8 mg/ml. During the 4-week expansion phase, colonoids were incubated in L-WRN medium (10% fetal bovine serum), which provides a source of Wnt3a, R-spondin-3, and Noggin. The medium was supplemented with small molecule inhibitors: 500nM A 83–01 (Tocris), a TGF-β inhibitor, 10μM SB 202190 (Sigma), a p38 inhibitor, and 10 μM Y27632 as a ROCK inhibitor (Tocris) [35]. For the first 10 days of culture, the medium was also supplemented with 2.5μM CHIR99021 (Tocris). In the absence of an enriched medium, normal colon crypts typically survive for only 3–5 days in culture [29], and this enriched medium was required for continued growth and maintenance of stem-like features [32,36]. For the experimental phase, established colonoids were interrogated in a mix of L-WRN culture medium diluted 1:4 with KGM Gold, a serum-free, calcium-free culture medium (Lonza). The final serum concentration was 2.5% and calcium concentration was 0.25 mM. This “control treatment medium” was compared to the same medium supplemented with calcium to a final concentration of 1.5–3.0 mM. Calcium was provided alone as calcium chloride or as Aquamin formulated to contain the equivalent amount of calcium. Colonoids from all subjects were evaluated by phase-contrast microscopy and histologically at the end of the 2-week treatment period. Due to the limited availability of crypts generated from small colon biopsies taken from each subject and expanded to test 6 culture conditions in addition to control (Calcium 0.25 mM), we strategized to perform proteomic analysis, immunohistochemistry and ultrastructural analysis with colonoids from at least 3 subjects.

### Phase-contrast microscopy

Colonoids were evaluated by phase-contrast microscopy (Hoffman Modulation Contrast—Olympus IX70 with a DP71 digital camera) for change in size and shape during the in-culture part of the study.

### Histology, immunohistology and morphometric analysis

At the end of the in-culture phase, colonoids were isolated from Matrigel using 2mM EDTA and fixed in 10% formalin for 1 hour. Fixed colonoids were suspended in HistoGel (Thermo Scientific) and then processed for histology (i.e., hematoxylin and eosin staining) or for immunohistology. For this, freshly-cut sections (5–6 microns) were rehydrated, and subjected to heat-induced epitope retrieval with high pH or low pH FLEX TRS Retrieval buffer (Agilent Technologies, 154 #K8004; Santa Clara, CA) for 20 minutes. After peroxidase blocking, antibodies were applied at appropriate dilutions at room temperature for 30 or 60 minutes (S2 Table). The FLEX HRP EnVision System (Agilent Technologies) was used for detection with a 10-minute DAB chromagen application. S2 Table provides a list of antibodies used and their source.

The sections of immunostained colonoid tissue on glass slides were digitized using the Aperio AT2 whole slide scanner (Leica Biosystems) at with a resolution of 0.5μm per pixel with 20x objective. These scanned images were housed on a server and accessed using Leica Aperio eSlide Manager (Version 12.3.2.5030), a digital pathology management software. The digitized histological sections were viewed and analyzed using Aperio ImageScope (Version 12.3.3.5048), a slide viewing software. Brightfield Immunohistochemistry Image Analysis tools (Leica) were used to quantify different immunostains used in this study. Aperio Nuclear Algorithm (v9) was used for proliferation marker (Ki67) quantification. This algorithm measures an intensity of the nuclear staining and separates those into very intense to no nuclear staining (3+, 2+, 1+ and 0 respectively). Nuclei with 3+ intensity were used here for comparison. The Aperio Positive Pixel Count Algorithm (v9) was used to quantify differentiation marker expression. It quantifies the number and the intensity of pixels of a specific stain in a digitized image. Positivity was calculated with respective numbers of strong positive and positive pixels against total pixels.

### Scanning electron microscopy (SEM) and transmission electron microscopy (TEM)

Colonoid specimens (control, calcium, and Aquamin at 1.5 mM concentration) were fixed in 2.5 percent glutaraldehyde in 0.1 M Sorensen’s buffer, pH 7.4, overnight at 4°C. After subsequent processing for SEM or TEM as described previously [37], samples for SEM were then mounted on stubs, allowed to off-gas in a vacuum desiccator for at least two hours and sputter coated with gold. Samples were examined with an Amray 1910 FE Scanning Electron Microscope and digitally imaged using Semicaps 2000 software. For TEM, ultra-thin sections were examined using a Philips CM100 electron microscope at 60 kV. Images were recorded digitally using a Hamamatsu ORCA-HR digital camera system operated with AMT software (Advanced Microscopy Techniques Corp., Danvers, MA).

### Differential proteomic analysis

Colonoids were isolated from Matrigel using 2mM EDTA for 15 minutes and then exposed to Radioimmunoprecipitation assay (RIPA)- lysis and extraction buffer (Thermo Scientific, Rockford, IL) for protein isolation. Proteomic experiments were carried out in the Proteomics Resource Facility (PRF) in the Department of Pathology at the University of Michigan, employing mass spectrometry-based Tandem Mass Tag (TMT, ThermoFisher Scientific). Fifty micrograms of colonoid protein from each condition (of each subject) were digested separately with trypsin and individually labeled with one of the 10 isobaric mass tags following the manufacturer’s protocol. After labeling, equal amounts of the peptide from each condition were mixed together. In order to achieve in-depth characterization of the proteome, the labeled peptides were fractionated using 2D-LC (basic pH reverse-phase separation followed by acidic pH reverse phase) and analyzed on a high-resolution, tribrid mass spectrometer (Orbitrap Fusion Tribrid, ThermoFisher Scientific) using conditions optimized at the PRF. MultiNotch MS3 approach [38] was employed to obtain accurate quantitation of the identified proteins/peptides. Data analysis was performed using Proteome Discoverer (v 2.1, ThermoFisher). MS2 spectra were searched against SwissProt human protein database (release 2016-11-30; 42054 sequences) using the following search parameters: MS1 and MS2 tolerance were set to 10 ppm and 0.6 Da, respectively; carbamidomethylation of cysteines (57.02146 Da) and TMT labeling of lysine and N-termini of peptides (229.16293 Da) were considered static modifications; oxidation of methionine (15.9949 Da) and deamidation of asparagine and glutamine (0.98401 Da) were considered variable. Identified proteins and peptides were filtered to retain only those that passed ≤2% false-discovery rate (FDR) threshold of detection. Quantitation was performed using high-quality MS3 spectra (Average signal-to-noise ratio of 9 and <40% isolation interference). Differential protein expression between conditions, normalizing to control (0.25mM calcium) for each subject’s specimens separately was established using edgeR [39]. Then, results for individual proteins from the three subjects were averaged. Proteins names were retrieved using Uniprot.org, and Reactome V66 (reactome.org) was used for pathway enrichment analyses [40]. Only proteins with a ≤2% false discovery rate (FDR) were included in the analyses. The initial analysis involved an unbiased proteome-wide screen of all proteins modified by Aquamin or by calcium alone in relation to control. Follow-up analysis was targeted towards differentiation, barrier-related, cell-cell and cell-matrix adhesion proteins. String database (string-db.org) was used to identify interactions between differentially-expressed proteins.

### Statistical analysis

Means and standard deviations were obtained for discrete morphological and immunohistochemical features as well as for individual protein values (proteomic analysis). Data generated in this way were analyzed by ANOVA followed by unpaired t-test (two-tailed) for comparison using GraphPad Prism version 8. Pathways enrichment data reflect Reactome-generated p-values based on the number of entities identified in a given pathway as compared to total proteins responsible for that pathway. Data were considered significant at p<0.05.

## Results

### Effects of calcium alone and Aquamin on structure of colonoids derived from histologically normal tissue

Histologically normal colon tissue was established in culture and incubated for a two-week period in control medium (0.25 mM calcium) or in the same culture medium supplemented with calcium to a final concentration of 1.5–3.0 mM or with Aquamin to provide the same levels of calcium. At the end of the incubation period, colonoids were examined for the gross appearance by phase-contrast and scanning electron microscopy and for microscopic appearance after sectioning and staining with hematoxylin and eosin.

**Fig 1:**
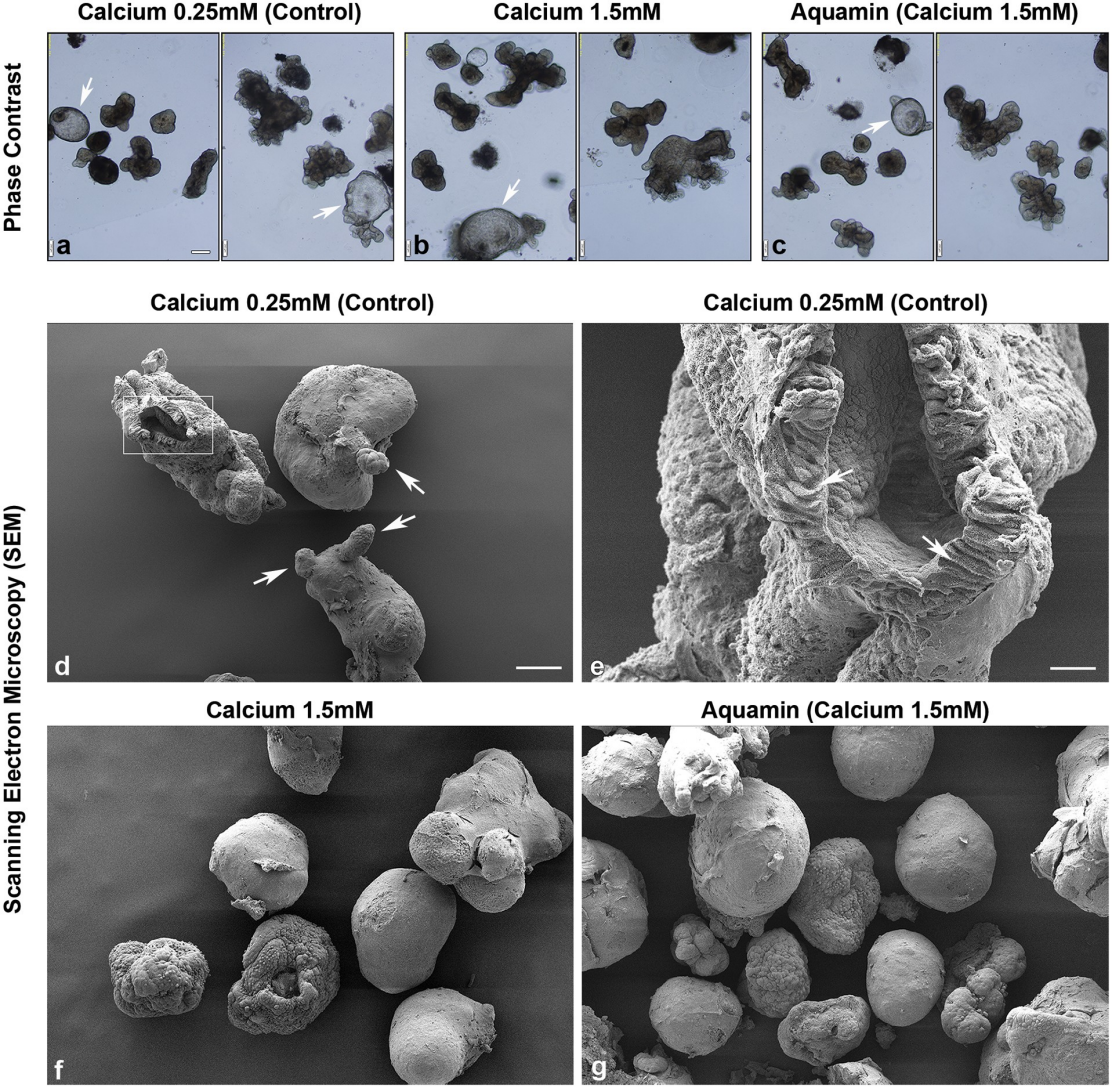
Colonoid appearance in culture: Phase-contrast and scanning electron microscopy. At the end of the incubation period, intact colonoids were examined. Under phase-contrast microscopy **(a-c)**, colonoids were present either as thin-walled, translucent cystic structures (arrows) or as thick-walled structures with few surface buds. They were similar in appearance under all conditions. Scanning electron microscopy confirmed the presence of smooth surface and few buds (arrows) in colonoids maintained under low-calcium conditions **(d)**. At higher magnification **(e)**, colonoids were shown to consist of hollow structures with a central lumen surrounded by columnar epithelial cells (arrows). Under high-calcium conditions **(f and g)**, colonoids were similar to those maintained in the low-calcium medium but without the surface buds. Bar for a, b and c = 200μm; bar for d, f and g = 100 μm; bar for e = 20 μm.

Representative phase-contrast microscopic images from day-14 cultures are shown in Fig 1a–1c. Colonoids maintained under control conditions (0.25 mM calcium) appeared as either thin-walled, translucent, cystic structures (arrows) that were mostly spherical or thick-walled structures with a variety of shapes (Fig 1a). When colonoids were maintained in the same medium but supplemented with calcium to a final concentration of 1.5 mM (provided either as calcium chloride or as Aquamin) (Fig 1b and 1c), there was minimal alteration in morphology. Both thin-walled, spherical structures and thick-walled structures were apparent, though we did not observe tiny buds growing out from the colonoid surface. Additional colonoids were treated with calcium (alone or as Aquamin) at 2.1 and 3.0 mM (S1 Fig). Colonoids maintained at these higher calcium levels were similar in appearance to those treated with 1.5 mM calcium. Cultures from all five subjects’ tissue were similar in appearance.

Scanning electron microscopy provided more precise evaluation of colonoid morphology (Fig 1d–1g). Under control (low-calcium) conditions (Fig 1d), colonoids had a spherical or oblong shape with a smooth surface. They were typically 100–200 μm in diameter. Most individual colonoids appeared to be “wrapped” with the matrix support, but in some cases, there was no visible matrix and the cells, themselves, formed the surface of the structure. Occasional small projections (buds) protruded from the colonoid surface (white arrows). In places, the colonoid surface was open (insert), allowing for visualization of the interior of the structure. At higher magnification (Fig 1e), it could be seen that the colonoid was hollow, with a central lumen surrounded by columnar epithelial cells (arrows). Colonoids maintained in the presence of 1.5 mM calcium (either alone or as Aquamin) (Fig 1f and 1g) were similar in appearance to the colonoids maintained in low-calcium medium. The one difference was that the small projections (buds) present in low-calcium colonoids were not seen at the higher calcium levels.

**Fig 2:**
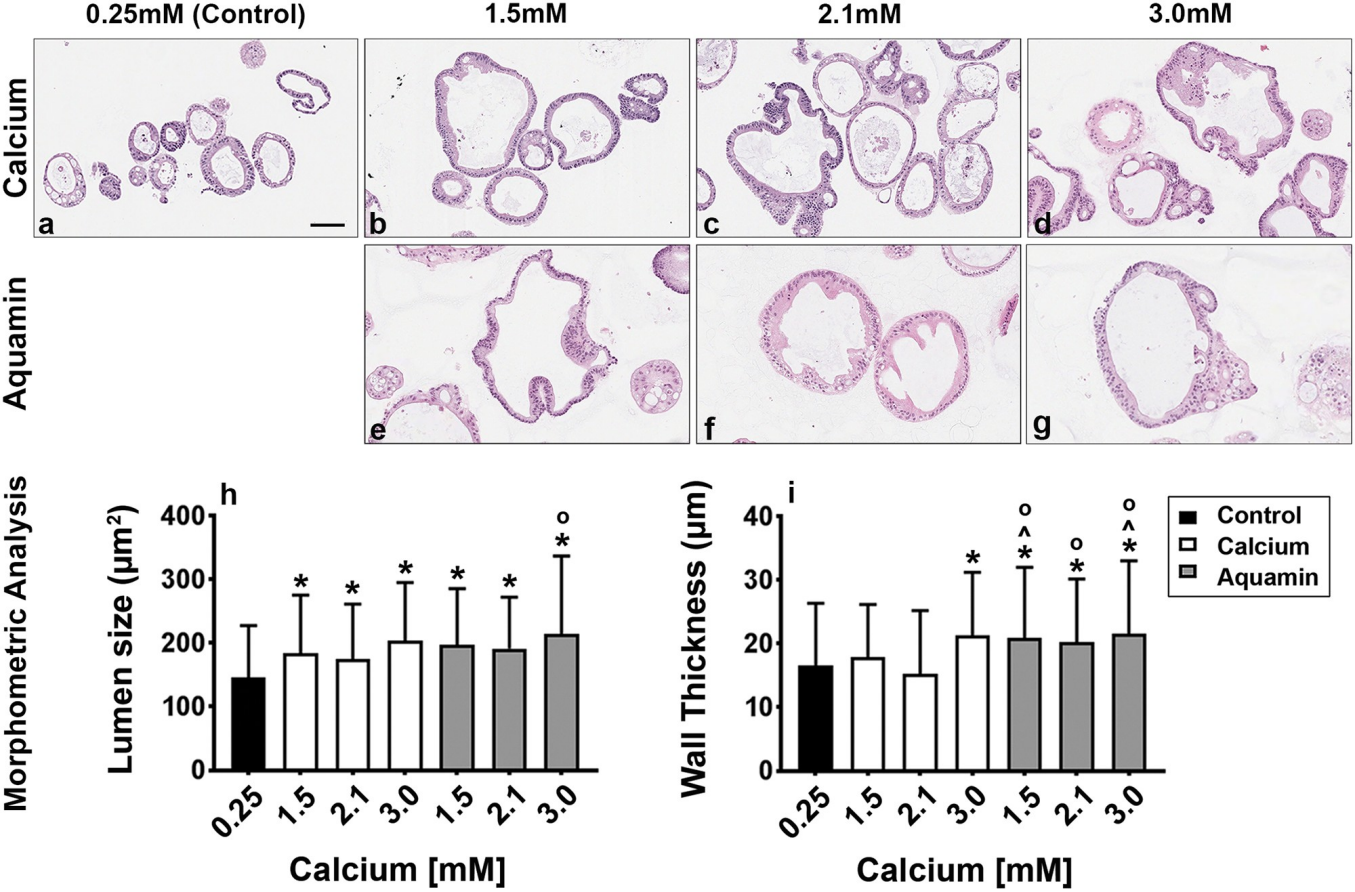
Colonoid appearance in culture: Histological features. At the end of the incubation period, colonoids were examined by light microscopy after staining with hematoxylin and eosin **(a-g)**. Under all three conditions, the colonoids were found to be crypts of varying size with a single layer of epithelial cells surrounding a central lumen. Under control conditions **(a)**, small crypts (with as few as 20 cells in cross section) were seen. In the presence of 1.5 mM calcium alone **(b)** or with Aquamin providing 1.5 mM calcium **(e)**, larger crypts made up of columnar epithelial cells surrounding a large, often irregular-in-shape lumen were seen. With higher calcium concentrations, either from calcium (**c** and **d**) or Aquamin (**f** and **g**), crypts morphology was similar to calcium or Aquamin at 1.5 mM—i.e., with an increase in the lumen size and wall thickness. Goblet cells were apparent. Lumen size and wall thickness **(h and i)** measurements are shown in the accompanying bar graphs. Means and standard deviations are based on 74–123 individual crypts per condition. Asterisks (*) indicate statistical significance from control. Additional symbols indicate statistical significance as follows: ^ from calcium 1.5mM, ^O^ from calcium 2.1mM, with a significance at p<0.05 level. Bar = 100μm.

Fig 2a–2g demonstrates microscopic appearance of colonoid sections after staining with hematoxylin and eosin, maintained under the same culture conditions. Under all conditions, colonoids were present as a mix of thin-walled crypts with spherical lumens, or as thick-walled crypts with lumens of varying shapes and sizes. The epithelial cells in the thin-walled crypts were mostly cuboidal in shape while the cells in the thick-walled structures were a mix of cells with cuboidal or columnar morphology. When microscopic features of hematoxylin and eosin stained sections were evaluated morphometrically, increases in both lumen diameter and wall thickness were observed under high-calcium conditions as compared to control (Fig 2h and 2i). Although, luminal diameter differences between colonoids exposed to calcium alone versus Aquamin were minimal, wall thickness was significantly greater with Aquamin (all conditions) as compared to calcium at 1.5 and 2.1 mM.

### Proliferation and differentiation marker expression patterns

**Fig 3:**
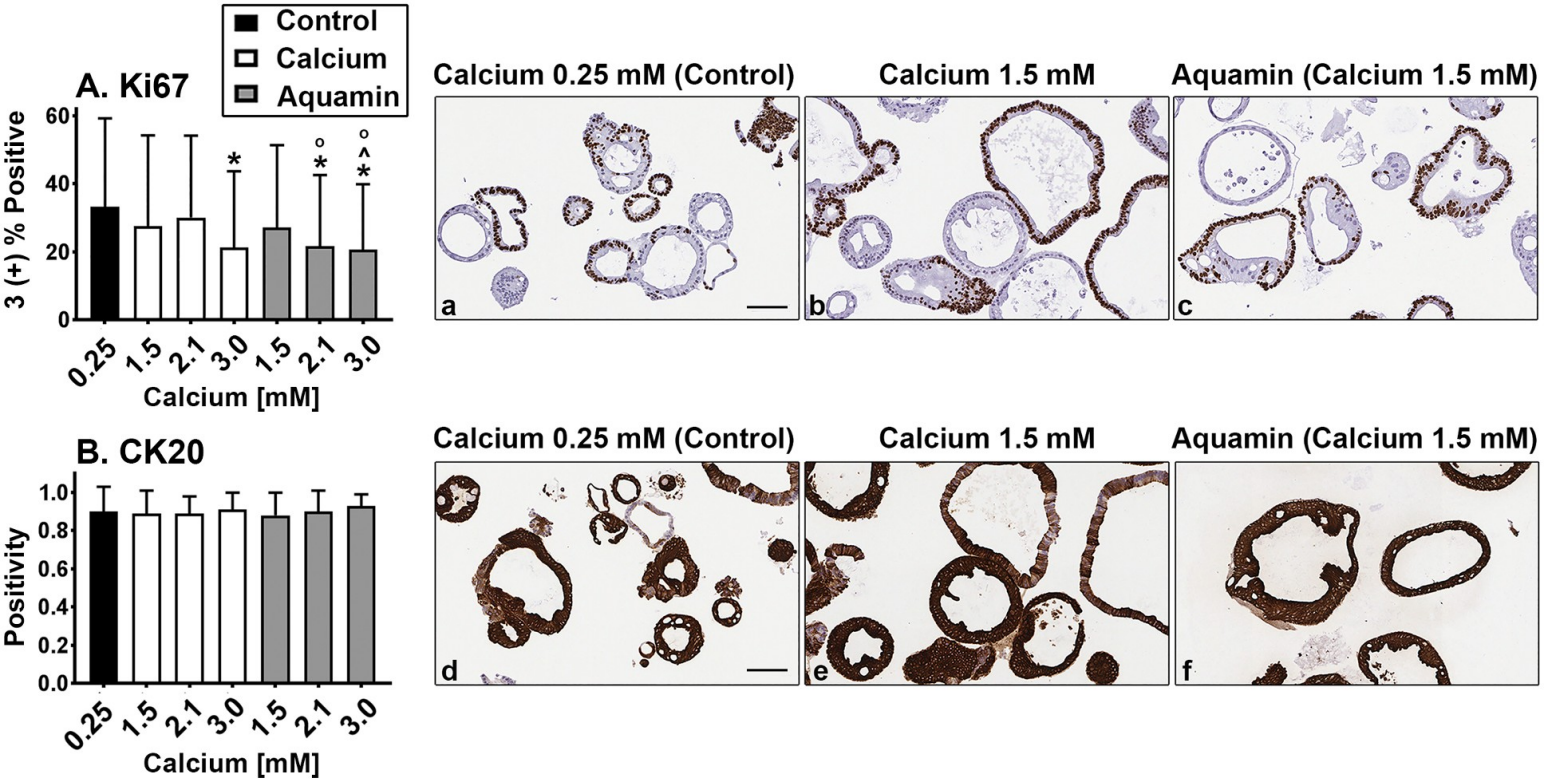
Proliferation and differentiation marker expression: Immunohistology. At the end of the incubation period, colonoids were examined after immunostaining. Ki67 expression (**A; a, b** and **c**). A mix of positive and negative staining was observed under all conditions. Morphometric analysis is shown in the accompanying bar graphs. Ki67 values (based on nuclear algorithm v9) are means and standard deviations based on 58–114 individual crypts per condition. Asterisks (*) indicate statistical significance from control. Additional symbols indicate statistical significance as follows: ^ from calcium 1.5mM, ^O^ from calcium 2.1mM, with a significance at p<0.05 level. CK20 expression (**B; d, e** and **f**). Most crypts, regardless of size or shape were strongly positive. CK20 values represent positivity (measured using Positive Pixel Value v9). Means and standard deviations based on 78–126 individual crypts per condition. Bars = 100μm.

Quantitative immunohistology was used to assess Ki67 expression as a proliferation marker and cytokeratin 20 (CK20) as a marker of epithelial cell differentiation (Fig 3). Ki67 expression was decreased modestly with intervention. The differences were statistically significant from control at 3.0 mM calcium and at 2.1 and 3.0 mM Aquamin. Aquamin at 2.1 mM was also significantly different from calcium at the comparable level (Fig 3A). CK20 was strongly positive in virtually all of the colonoids regardless of treatment (Fig 3B). Additional images showing proliferation (Ki67) / differentiation (CK20) marker expression at higher ambient calcium levels are shown in S2 Fig.

### Proteomic analysis: Up-regulation of proteins involved in differentiation and related functions

**Fig 4:**
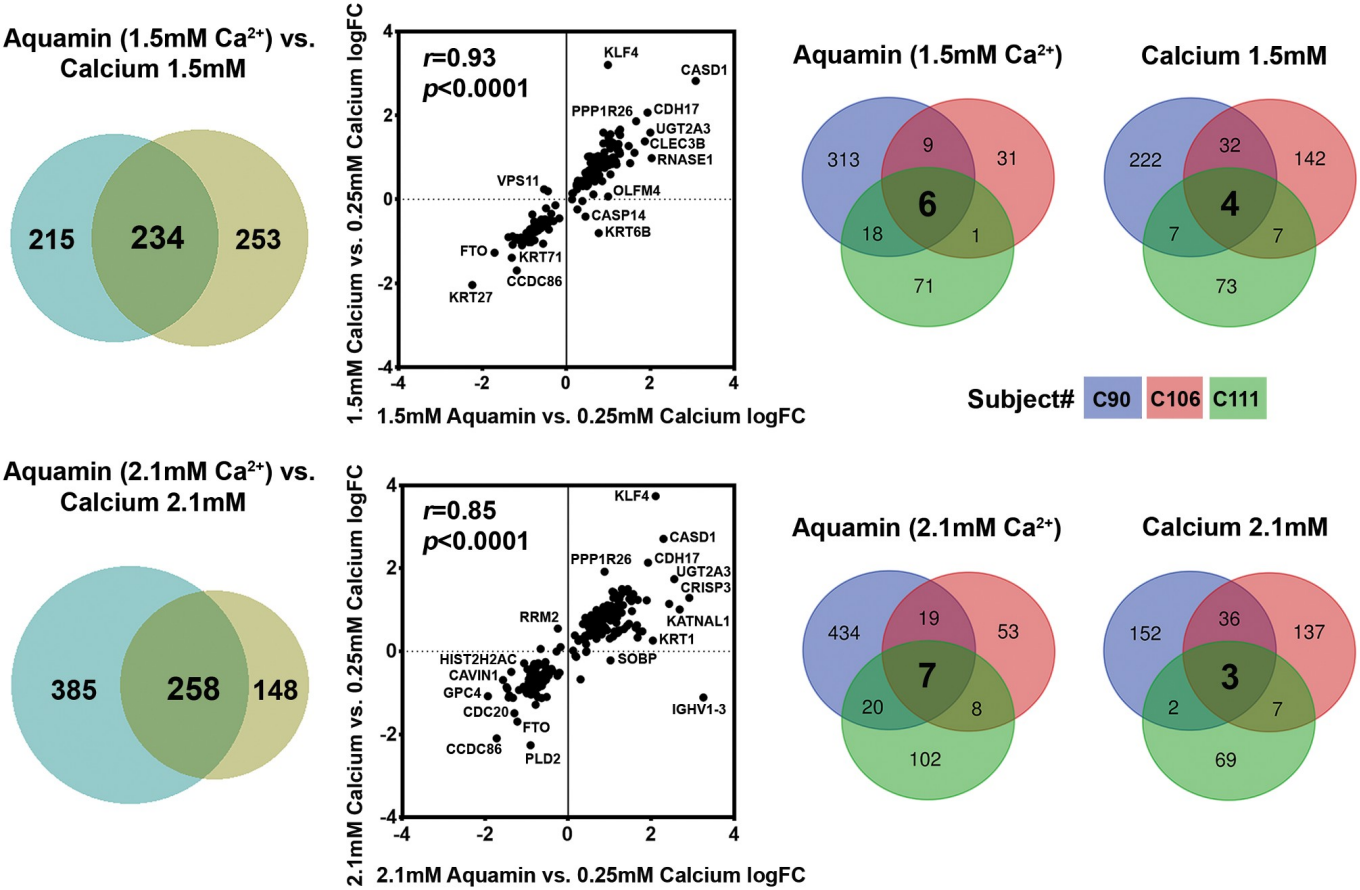
Proteomic analysis of human colonoids. At the end of the incubation period, lysates were prepared for proteomic analysis. **Left**: Venn plots showing proteins altered (increased or decreased) by an average of 1.8-fold or greater across the three specimens in response to calcium alone or to Aquamin with a comparable amount of calcium, and the overlap between pairs of interventions. **Center**: The scatterplots demonstrate quantitative relationships between individual proteins altered by each pair of interventions. Correlation between the two interventions at both concentrations was significant (p<0.0001). **Right**: Venn plots showing proteins altered (increased or decreased) by an average of 1.8-fold or greater with each intervention and the overlap among individual specimens. This is to show the variability in subjects and a response to the intervention.

We utilized a mass spectrometry-based approach to identify differentiation-related proteins expressed in colonoid cultures (from 3 subjects) and assessed the effects of calcium alone and Aquamin on expression levels relative to individual control conditions. For an initial assessment, a non-biased search of all proteins up-regulated or down-regulated by either intervention was conducted. Average increase or decrease values across the three specimens (representing proteins altered by 1.8-fold or greater with <2% FDR in at least one specimen) are presented in Fig 4. The Venn plots shown in the left-portion of Fig 4 demonstrate a substantial concurrence between proteins up- or down-regulated by the two interventions at comparable calcium levels and the scatter plots in the center show the relationship between the degree of up- or down-regulation in the overlapping proteins. The Venn plots shown in the right-hand portion of Fig 4 demonstrate the distribution of proteins among the three specimens (subjects). Interestingly, only one protein—cadherin-17 —met the criterion of being increased by greater than 1.8-fold with both interventions at all concentrations in all three specimens.

Several individual proteins of interest were identified with an unbiased approach. Up-regulated proteins are presented in Table 1 and down-regulated proteins are shown in Table 2). Among up-regulated proteins were several that are directly related to differentiation (i.e., a number of keratins and hornerin) as well as proteins involved in cell-cell or cell-matrix adhesion (cadherin-17, protocadherin-1, CEACAM 7 and desmoglein-2). Also up-regulated was vitamin D-binding protein, the major transporter of vitamin D in the circulation. This protein is responsible for carrying vitamin D into cells [41] and is, therefore, critical to calcium uptake and utilization at the cellular level. Intestinal alkaline phosphatase and 15-hydroxyprostaglandin dehydrogenase were two additional proteins of interest that were substantially up-regulated. Elevated expression of these proteins is associated with inflammation-suppression in the gastrointestinal tract [42–45]. They provide potential links between improved barrier function and decreased inflammation. Among down-regulated moieties of interest were proteins related to growth—i.e., proliferating cell nuclear antigen (PCNA) [46] and nucleophosmin (NPM) [47,48]. S3A and S3B Table present pathways associated with the up-regulated and down-regulated proteins. A number of differentiation-related pathways as well as pathways associated with cell-cell adhesion, cell-substrate adhesion and barrier function were identified in response to the up-regulated proteins.

**Table 1:** Up-regulated proteins (unbiased approach).

Proteins	Calcium	Calcium	Calcium	Aquamin	Aquamin	Aquamin
	1.5mM	2.1mM	3.0mM	(1.5mM Calcium)	(2.1mM Calcium)	(3.0mM Calcium)
**Keratin, type I cytoskeletal 9**	1.07±0.47	1.79±1.73	*0.59±0.13	3.66±4.03	9.96±15.39	0.98±0.34
**Keratin, type II cytoskeletal 1**	0.94±0.30	1.58±1.41	0.73±0.34	2.79±3.22	7.12±8.56	0.95±0.12
**Keratin, type I cytoskeletal 16**	0.98±0.14	3.88±4.88	1.00±0.14	5.59±8.08	8.11±7.19	0.92±0.22
**Hornerin**	1.68±1.22	1.82±1.28	0.76±0.09	1.99±0.95	4.98±6.49	0.94±0.62
**Keratin, type II cytoskeletal 6A**	0.92±0.16	1.80±1.59	0.98±0.07	3.95±5.37	4.95±5.38	1.07±0.25
**Keratin, type I cytoskeletal 14**	0.84±0.25	3.33±3.45	1.24±0.71	1.88±1.89	4.36±2.68	0.99±0.14
**Keratin, type II cytoskeletal 5**	1.14±0.34	1.47±0.60	1.08±0.15	2.83±3.09	4.50±3.04	0.98±0.02
**Cadherin-17**	*4.28±1.04	*4.57±1.43	*5.65±0.69	*3.89±0.90	*3.87±0.98	*4.62±0.25
**Vitamin D-binding protein**	2.99±1.28	3.29±2.50	3.49±2.12	*2.04±0.49	2.27±0.85	1.75±0.56
**Carbonic anhydrase 1**	1.70±0.51	1.83±0.73	*1.96±0.17	2.16±1.97	2.10±1.87	1.02±0.29
**Lactotransferrin**	3.08±1.81	3.09±1.86	*1.94±0.15	2.33±1.10	2.74±1.52	1.74±0.88
**Sulfate transporter**	2.54±1.04	2.31±1.02	*2.20±0.15	3.19±2.09	2.96±2.14	*1.91±0.20
**Zinc transporter ZIP4**	2.28±1.51	2.00±1.04	1.51±0.86	2.30±0.90	2.45±1.56	1.68±0.78
**Natural resistance-associated macrophage protein 2**	2.45±1.44	2.06±0.70	*1.65±0.20	*2.48±0.80	2.58±1.49	*2.27±0.13
**Alpha-2-HS-glycoprotein**	2.57±1.07	2.76±1.19	2.82±0.86	*2.10±0.6	2.42±1.36	1.45±0.42
**Aminopeptidase N**	*2.31±0.56	*2.05±0.52	*2.40±0.32	2.46±0.93	2.28±1.27	1.68±0.37
**Prostate stem cell antigen**	*2.50±0.64	*2.20±0.72	*2.55±0.15	2.27±0.97	2.08±1.41	1.59±0.94
**Xaa-Pro aminopeptidase 2**	*1.91±0.55	1.84±0.63	1.47±0.26	2.38±0.96	2.40±1.09	1.64±0.36
**Intestinal-type alkaline phosphatase**	*2.33±0.76	*2.16±0.54	*2.29±0.26	*2.38±0.56	2.35±0.98	1.82±0.70
**Alpha-2-macroglobulin**	*2.55±0.39	*2.48±0.78	*2.80±0.15	*2.18±0.14	*2.79±0.40	*2.70±0.005
**Meprin A subunit alpha**	*2.07±0.65	*2.01±0.53	*2.13±0.17	2.30±0.83	2.01±1.14	1.51±0.76
**Chloride anion exchanger**	*1.94±0.47	*1.81±0.42	*1.87±0.11	2.23±0.95	2.05±1.00	1.56±0.51
**CEACAM7**	*2.05±0.52	*1.92±0.46	*1.94±0.04	2.26±0.88	2.13±0.93	1.59±0.40
**Desmoglein-2**	*2.52±0.42	*2.54±0.61	*3.15±0.06	*2.40±0.52	*2.37±0.57	*2.70±0.07
**Complement C3**	*2.58±0.36	*2.41±0.65	*2.71±0.56	*2.00±0.28	*2.38±0.71	*1.89±0.11
**Trefoil factor 2**	*1.83±0.33	*1.91±0.25	*1.93±0.28	1.95±0.94	1.75±0.84	1.45±0.64
**Protocadherin-1**	*2.21±0.43	*2.48±0.52	2.37±0.84	*1.90±0.38	*2.13±0.18	*2.17±0.25
**15-hydroxyprostaglandin dehydrogenase [NAD(+)]**	1.90±0.61	*1.91±0.53	*2.03±0.27	1.91±0.71	1.96±0.83	1.42±0.22
**Solute carrier family 15 member 1**	1.91±0.57	1.68±0.46	*1.95±0.14	1.95±0.71	1.85±0.89	1.36±0.34
**Calcium-activated chloride channel regulator 4**	*1.77±0.43	*1.75±0.31	*2.07±0.13	1.87±0.79	2.00±0.82	1.55±0.63
**Hydroxymethylglutaryl-CoA synthase, mitochondrial**	*1.78±0.37	*1.82±0.39	*1.88±0.11	*1.88±0.38	*1.99±0.16	*2.07±0.13
**Pantetheinase**	1.46±0.35	*1.39±0.24	*1.76±0.14	1.40±0.38	1.27±0.50	1.17±0.15

Values represent average fold change from 3 colonoids as compared to control (0.25 mM calcium) ±SD. These proteins were up-regulated in all 3 colonoids at 1.8-fold change and were common in all three colonoids based on a maximum up-regulation in at least one condition. Some of the common up-regulated proteins have presented in Table 3 as part of the differentiation-related panel.

*Represents significance as compared to the control at p<0.05.

**Table 2:** Down-regulated proteins (unbiased approach).

Proteins	Calcium	Calcium	Calcium	Aquamin	Aquamin	Aquamin
	1.5mM	2.1mM	3.0mM	(1.5mM Calcium)	(2.1mM Calcium)	(3.0mM Calcium)
**Coiled-coil domain-containing protein 86**	*0.377±0.266	*0.317±0.247	*0.255±0.143	*0.462±0.157	*0.341±0.174	*0.279±0.144
**Caveolae-associated protein 1**	*0.551±0.174	0.807±0.684	*0.445±0.074	*0.455±0.139	*0.386±0.247	*0.451±0.019
**Proliferating cell nuclear antigen**	0.677±0.279	0.796±0.435	*0.485±0.146	*0.608±0.134	*0.556±0.099	*0.605±0.069
**Inosine-5'-monophosphate dehydrogenase 2**	*0.598±0.209	*0.614±0.170	*0.453±0.040	*0.523±0.081	*0.526±0.126	*0.487±0.076
**Importin subunit alpha-1**	0.785±0.415	0.922±0.707	*0.515±0.054	*0.674±0.159	*0.566±0.150	0.621±0.150
**60S ribosomal protein L36**	*0.538±0.054	*0.551±0.132	*0.559±0.087	*0.769±0.080	*0.564±0.073	*0.654±0.017
**RNA-binding protein with serine-rich domain 1**	*0.708±0.057	*0.643±0.120	*0.476±0.060	*0.781±0.124	0.742±0.190	0.819±0.094
**Nucleophosmin**	*0.590±0.104	0.714±0.255	0.607±0.161	*0.603±0.081	*0.541±0.097	*0.590±0.076

Values represent average fold change from 3 colonoids as compared to control (0.25 mM calcium) ±SD. These proteins were down- regulated in all 3 colonoids at 1.8-fold change and were common in all three colonoids based on a maximum down-regulation in at least one condition.

*Represents significance as compared to the control at p<0.05.

As colonic mucosal differentiation in response to either calcium alone or Aquamin was the primary focus of the study, we looked specifically for proteins involved in differentiation *per se*, as well as for proteins involved in cell-cell and cell-matrix adhesion and barrier function. Table 3 is a compilation of such proteins and Table 4 presents the pathways associated with these proteins. Average fold-change values across the three specimens with each intervention at day-14 are compared to control conditions. As can be seen from the Table 3, there was an increase in CK20 expression along with an increased level of epiplakin—a protein required for intermediate filament network formation and differentiation [49]. These are in addition to the keratins identified in the unbiased screen (S4 Table).

**Table 3:** Differentiation-related proteins.

Proteins	Calcium	Calcium	Calcium	Aquamin	Aquamin	Aquamin
	1.5mM	2.1mM	3.0mM	(1.5mM Calcium)	(2.1mM Calcium)	(3.0mM Calcium)
**Differentiation-related**						
**Keratin, type I cytoskeletal 20 (CK20)**	1.42±0.33	*1.46±0.21	*1.62±0.06	1.42±0.33	*1.47±0.24	1.35±0.15
**Epiplakin**	1.57±0.73	1.42±0.47	*1.39±0.11	1.43±0.39	1.57±0.58	*1.76±0.35
**Cadherin-family members**						
**Cadherin-17**	*4.28±1.04	*4.57±1.43	*5.65±0.69	*3.89±0.9	*3.87±0.98	*4.62±0.25
**Protocadherin-1**	*2.21±0.43	*2.48±0.52	2.37±0.84	*1.90±0.38	*2.13±0.18	*2.17±0.25
**Cadherin-related family member 2**	1.63±0.57	1.57±0.41	*1.52±0.02	1.46±0.51	1.53±0.65	1.13±0.33
**Cadherin-related family member 5**	1.66±0.56	1.54±0.41	1.45±0.24	1.56±0.60	1.60±0.67	1.25±0.30
**Cadherin-1**	1.01±0.07	1.05±0.03	1.01±0.10	1.02±0.10	0.98±0.09	0.92±0.01
**Tight junctional proteins**						
**Claudin-3**	1.23±0.21	1.08±0.11	1.11±0.09	1.14±0.16	1.12±0.15	1.12±0.27
**Claudin-4**	1.22±0.23	1.17±0.23	*1.17±0.05	1.18±0.14	1.08±0.22	*1.14±0.03
**Claudin-7**	1.24±0.35	1.25±0.27	1.22±0.25	1.28±0.28	1.10±0.34	1.17±0.12
**Claudin-23**	3.46±3.84	2.34±1.87	*1.36±0.07	3.05±2.80	2.04±1.77	1.36±0.71
**Occludin**	1.08±0.15	1.12±0.13	1.01±0.10	1.06±0.15	1.03±0.14	0.96±0.10
**Desmosomal proteins**						
**Desmoglein-2**	*2.52±0.42	*2.54±0.61	*3.15±0.06	*2.40±0.52	*2.37±0.57	*2.70±0.07
**Desmocollin-2**	*1.84±0.18	*1.87±0.25	*2.11±0.32	*1.81±0.24	*1.68±0.17	*1.81±0.07
**Desmoplakin**	1.64±0.62	1.39±0.30	1.59±0.26	1.52±0.50	1.75±0.73	1.83±0.52
**PERP**	*1.75±0.26	*1.61±0.22	1.78±0.32	*1.51±0.24	*1.62±0.32	1.56±0.21
**Cell-matrix adhesion proteins**						
**Laminin subunit alpha-1**	1.46±0.41	1.40±0.43	*2.78±0.12	1.62±0.44	2.59±1.61	*3.54±0.76
**Laminin subunit beta-1**	1.50±0.51	1.47±0.53	*2.93±0.01	1.70±0.47	2.66±1.87	*3.66±0.65
**Laminin subunit beta-2**	1.26±0.20	*1.37±0.18	2.62±1.21	1.64±0.69	*1.90±0.50	3.50±2.33
**Laminin subunit gamma-1**	1.54±0.54	1.49±0.57	*3.11±0.10	1.71±0.50	2.81±2.07	*3.97±0.94
**CEACAM1**	1.81±0.52	*1.61±0.35	*1.61±0.03	1.88±0.74	3.91±3.69	1.25±0.32
**CEACAM5**	1.43±0.29	*1.36±0.19	1.51±0.22	*1.52±0.31	1.54±0.61	1.24±0.56
**CEACAM6**	*1.26±0.15	1.20±0.14	*1.26±0.01	1.33±0.32	1.37±0.47	1.01±0.23
**CEACAM7**	*2.05±0.52	*1.92±0.46	*1.94±0.04	2.26±0.88	2.13±0.93	1.59±0.40
**Nidogen-1**	1.53±0.51	1.45±0.50	*2.68±0.27	*1.58±0.24	2.53±1.82	*3.24±0.002
**Fibronectin type III domain 3B**	1.72±0.99	1.52±0.70	1.13±0.28	1.32±0.34	2.10±1.47	1.27±0.26
**HSPG2 (Perlecan)**	1.18±0.12	1.20±0.17	*1.49±0.25	1.18±0.15	1.44±0.49	*1.63±0.14
**Calcium and integrin-binding protein 1**	*1.26±0.12	*1.30±0.10	*1.38±0.08	*1.36±0.14	*1.24±0.12	1.21±0.14
**Integrin alpha-V**	1.22±0.15	*1.23±0.14	*1.21±0.07	*1.19±0.10	1.19±0.13	1.19±0.07
**Integrin beta-5**	1.27±0.28	1.17±0.11	*1.07±0.02	1.13±0.08	1.23±0.18	*1.11±0.01
**CD44 antigen**	0.69±0.25	0.77±0.35	0.67±0.28	*0.60±0.09	*0.56±0.11	*0.54±0.03
**Mucin-5B**	1.51±0.41	*1.54±0.35	*1.55±0.19	*1.49±0.23	1.62±0.57	1.17±0.42
**Other proteins of interest**						
**Olfactomedin-4**	1.36±0.91	1.39±0.63	1.22±0.93	2.16±1.00	*2.14±0.43	2.27±1.54
**Transcriptional coactivator YAP1**	0.58±0.35	0.68±0.29	0.49±0.45	0.71±0.22	*0.72±0.12	*0.73±0.14
**Spindlin-1**	0.77±0.32	*0.72±0.07	*0.59±0.04	0.77±0.22	0.66±0.32	0.81±0.42

Values represent average fold-change across specimens from three different subjects. Specimens from each subject were assessed separately and fold-change with each intervention compared to control (0.25 mM calcium). Then values from each intervention for all three subjects were averaged to generate the average fold-change values shown. Asterisks indicate statistical significance at p<0.05 based on student t-test with correction for multiple comparisons. PERP = P53 apoptosis effector related to PMP22. CAECAM = Carcinoembryonic antigen-related cell adhesion molecule. HSPG2 = Heparan Sulfate Proteoglycan 2.

**Table 4:** Top pathways involved with proteins presented in Table 3.

Pathway name	Entities p Value	Mapped entities
**Laminin interactions**	6.7x10^-12^	LAMB1;ITGAV;LAMB2;LAMA1;HSPG2;LAMC1;NID1
**Extracellular matrix organization**	7.4x10^-11^	LAMB1;CEACAM6;ITGAV;LAMB2;LAMA1;ITGB5;CDH1; CEACAM1;HSPG2;LAMC1;NID1; CD44
**Non-integrin membrane-ECM interactions**	7.1x10^-10^	LAMB1;ITGAV;LAMB2;LAMA1;ITGB5;HSPG2;LAMC1
**ECM proteoglycans**	4.0x10^-9^	LAMB1;ITGAV;LAMB2;LAMA1;ITGB5;HSPG2;LAMC1
**Apoptotic cleavage of cell adhesion proteins**	4.9x10^-8^	DSG2;OCLN;DSP;CDH1
**Cell-cell junction organization**	5.7x10^-8^	CLDN23;CLDN3;CLDN4;CDH17;CLDN7;CDH1
**Cell junction organization**	4.3x10^-7^	CLDN23;CLDN3;CLDN4;CDH17;CLDN7;CDH1
**Tight junction interactions**	2.6x10^-6^	CLDN23;CLDN3;CLDN4;CLDN7
**Cell-Cell communication**	3.1x10^-6^	CLDN23;CLDN3;CLDN4;CDH17;CLDN7;CDH1
**Apoptotic cleavage of cellular proteins**	6.6x10^-6^	DSG2;OCLN;DSP;CDH1
**Integrin cell surface interactions**	7.1x10^-6^	ITGAV;ITGB5;CDH1;HSPG2; CD44
**Apoptotic execution phase**	2.3x10^-5^	DSG2;OCLN;DSP;CDH1
**Formation of the cornified envelope**	5.2x10^-5^	DSG2;DSC2;DSP;KRT20;PERP
**Fibronectin matrix formation**	1.7x10^-4^	CEACAM6;CEACAM1
**Keratinization**	5.7x10^-4^	DSG2;DSC2;DSP;KRT20;PERP
**L1CAM interactions**	5.7x10^-4^	LAMB1;ITGAV;LAMA1;LAMC1
**Apoptosis**	0.002	DSG2;OCLN;DSP;CDH1
**Programmed Cell Death**	0.002	DSG2;OCLN;DSP;CDH1
**Syndecan interactions**	0.003	ITGAV;ITGB5
**Adherens junctions interactions**	0.005	CDH17;CDH1
**Developmental Biology**	0.005	LAMB1;DSG2;ITGAV;DSC2;LAMA1;DSP;KRT20;PERP;LAMC1
**RUNX1 regulates expression of components of tight junctions**	0.02	OCLN
**PECAM1 interactions**	0.04	ITGAV
**TP53 regulates transcription of cell death genes**	0.04	PERP

Reactome database (v66) and Uniprot are the source of information.

Several proteins that make up adherens junctions (cadherin family members) and desmosomes (desmoglein-2, desmocollin-2, desmoplakin and p53 apoptosis effector related to PMP-22 [PERP]) were strongly and consistently up-regulated (Table 3). In contrast, tight junction protein up-regulation was more modest. Only claudin-23 was substantially increased, and this was due, primarily, to up-regulation in one specimen (Table 3). Table 3 also demonstrates that several proteins involved in cell-matrix adhesion were substantially elevated in samples treated with calcium alone or Aquamin as compared to control. Among these were laminin subunits, several carcinoembryonic antigen-related cell adhesion molecules (CEACAMs), nidogen, fibronectin type III domain protein and perlecan (basement membrane specific heparin sulfate proteoglycan-2). Integrin alpha V and integrin beta 5 subunits were also up-regulated with the two interventions (Table 3) while virtually all of the other alpha and beta integrin subunits showed little difference with calcium alone or with Aquamin treatment (S4 Table). In contrast to these up-regulated proteins, CD44, a hyaluronan receptor associated with multiple cell functions including cell motility was down-regulated with both interventions, but more strongly with Aquamin (Table 3). High CD44 expression has been linked to cancer metastasis [50].

Table 4 highlights the top 24 pathways associated with the proteins presented in Table 3. Not surprisingly, most of these pathways involve the extracellular matrix, cell-cell junctional organization and cell-cell communication. Known interactions among the proteins listed in Table 3 are presented using String database (S3 Fig). As seen, the cell-cell and cell-matrix adhesion proteins cluster together. These clusters interact with each other through cadherins (primarily cadherin 17).

Finally, we searched the list of up-regulated and down-regulated proteins for other moieties of interest that had been shown in our previous study [33] to be strongly affected by calcium alone or Aquamin in colonoid cultures established from large, premalignant adenomas. Among the proteins of interest identified in adenomas were moieties associated with growth-regulating pathways; i.e. NF2 (merlin), BRCA-related protein, members of the histone 1 family and olfactomedin-4. Among down-regulated proteins were metallothionine 1E and 1H. Of these, only olfactomedin-4 was sufficiently abundant to reach threshold level in normal colonoids. It was modestly up-regulated by calcium alone, but more highly induced by Aquamin (1.22–1.39-fold increase with calcium—not significant; and 2.14–2.27-fold with Aquamin; p<0.05) (Table 3). Olfactomedin-4 is of interest because while it is clearly responsive to differentiation-inducing interventions, it is also expressed in cells in concurrence with other stem cell markers [51]. Also of interest, two transcription enhancers associated with dysregulated growth (YAP-1 and spindlin-1) [52,53] were both down-regulated with calcium and Aquamin (Table 3). YAP-1 is a downstream target in the HIPPO signaling pathway. NF2, a potent inhibitor of HIPPO signaling, was strongly up-regulated by Aquamin in adenoma colonoids [33].

### Immunohistological and ultrastructural assessment of barrier proteins

**Fig 5:**
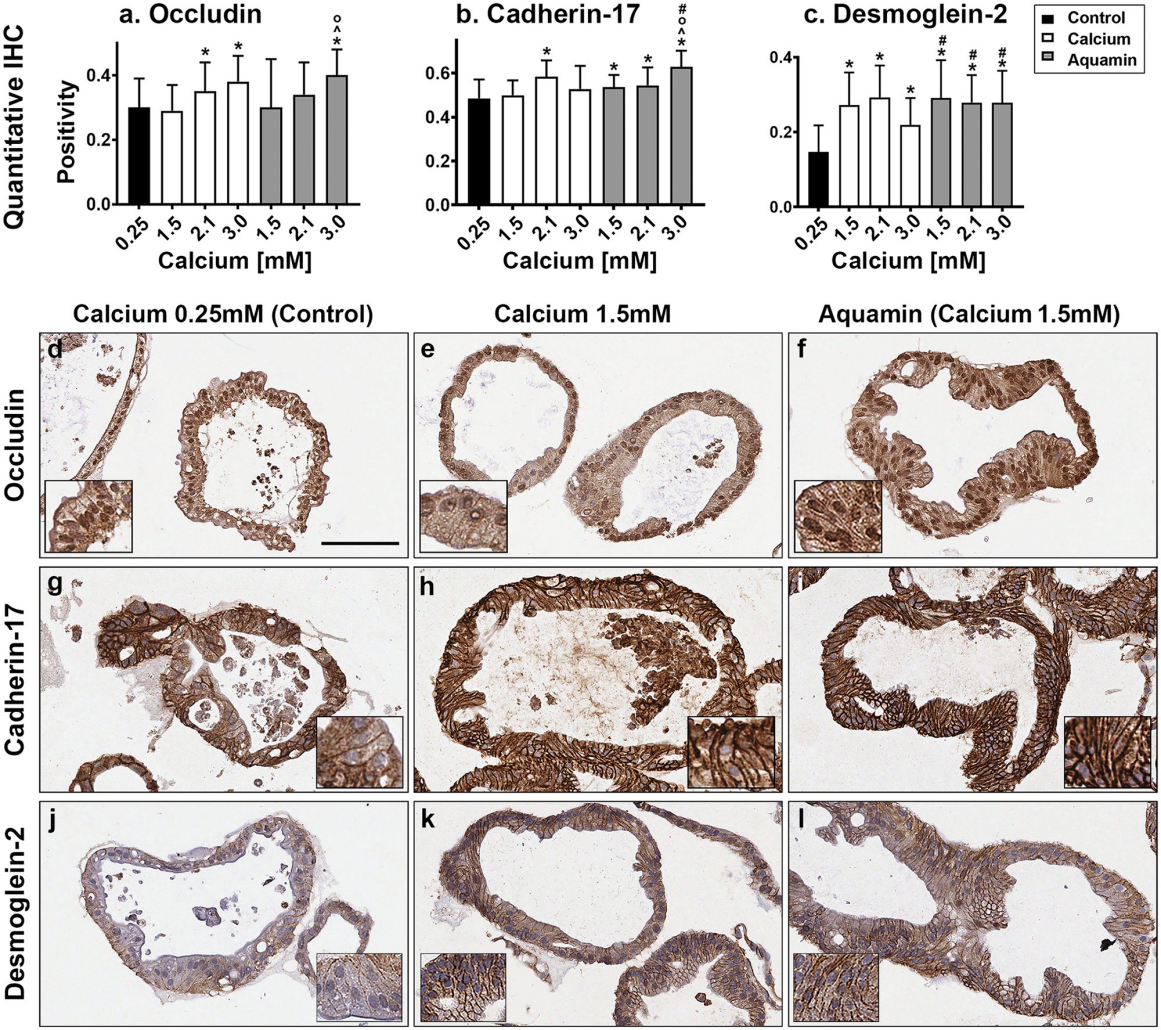
Cell surface components. At the end of the incubation period, colonoids were examined after immunostaining of histological sections. Quantitative immunohistochemical analysis (**a-c**) and images: occludin **(d, e and f)**; Cadherin-17 **(g, h and i)**; desmoglein-2 **(j, k and l).** Quantitative IHC. Positivity measured using Positive Pixel Value algorithm. Means and SDs based on 26 to 100 individual crypts in each condition. Asterisks (*) indicate statistical significance from control. Additional symbols indicate statistical significance as follows: ^ from calcium 1.5mM, ^O^ from calcium 2.1mM, and # from calcium 3.0mM, with a significance at p<0.05 level. All three proteins are visible in colonoids under all conditions by immunohistology. With desmoglein-2, there is a clear shift from cytoplasmic to surface with intervention. Bar for (d-l) main panel = 100μm. Insets: respective stained colonoids at a higher resolution.

Based on proteomic findings, we chose three proteins—occludin (tight junction), cadherin-17 (adherens junction), and desmoglein-2 (macula adherens or desmosomes)–for assessment using quantitative immunohistology. All three proteins were expressed under control conditions (Fig 5a–5c); a mixture of cytoplasmic and cell surface staining was seen (Fig 5d, 5g and 5j). Increased expression of all three proteins was observed in response to calcium alone or Aquamin (Fig 5a–5c). With occludin and cadherin-17, increased expression was seen only at the higher levels of intervention (2.1 and 3.0 mM) but with desmoglein-2, a substantial and statistically significant increase was observed at concentrations of either intervention delivering 1.5 mM calcium (or higher).

Representative images from control colonoid cultures and cultures incubated with 1.5 mM calcium alone or with Aquamin are shown along with the quantitative findings. With occludin (Fig 5d–5f), the images show a mixture of cytoplasmic and cell surface staining. Little change with intervention is observed at the lower concentrations. With cadherin-17, a modest shift from cytoplasmic to cell surface can be seen with either intervention (Fig 5g–5i). In contrast, there is a strong shift from cytoplasmic to the cell surface with desmoglein-2 (Fig 5j–5l) with either intervention. S2 Fig shows additional images of the same three proteins with higher concentrations of calcium and Aquamin. With both cadherin-17 and desmoglein-2, strong surface staining is seen.

**Fig 6:**
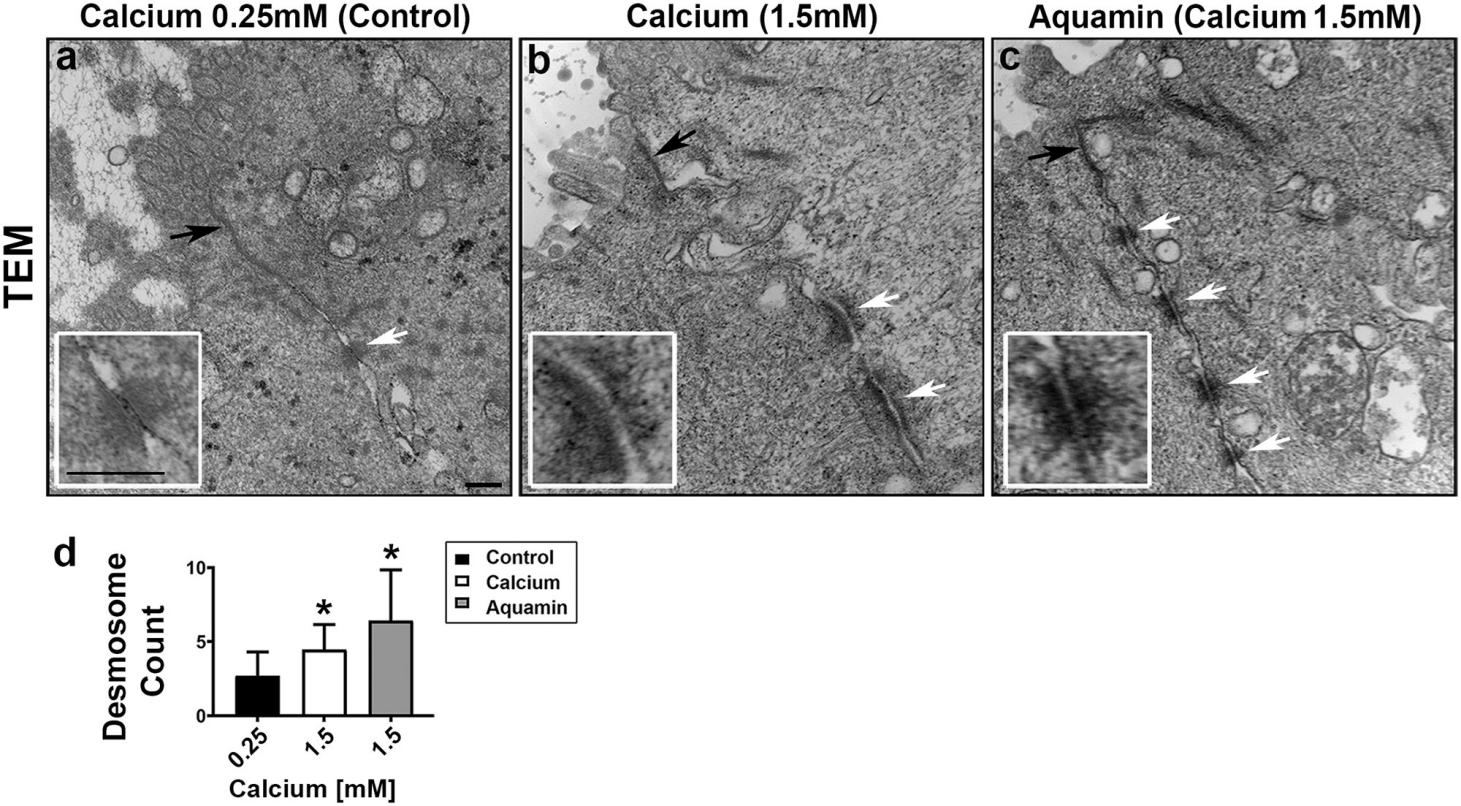
Ultrastructure by transmission electron microscopy. At the end of the incubation period, colonoids were examined for ultrastructures using TEM **(a-c)**. Under all conditions (a-c), tight junctions were evident right below the epithelial layer on the luminal side (black arrows). Desmosomes were present in all conditions (white arrows) but a higher density of desmosomes along the lateral surface (cellular junctions between two cells) can be seen with intervention (at 10,000X). Bars = 200 nm (Insets: Desmosomes in close up). The desmosome count (d) was conducted at 5000X (high-power fields; n = 9–18) to get the actual number (means and SD) of desmosomes present in each high-power section. Asterisks indicate statistical significance from control at p<0.05 level. The p-value was equal to 0.0529 for the comparison between calcium and Aquamin.

In a final set of studies, colonoid specimens from three subjects were examined by transmission electron microscopy. The focus was on regions of cell-cell contact. Electron microscopy demonstrated differences in desmosome (white arrows) expression in response to intervention as compared to control (Fig 6a–6c). Under low-calcium (control) conditions, desmosomes were few in number, size and electron density. We rarely saw more than a single desmosome in any high-power (10,000X) image. In contrast, colonoids maintained in medium containing 1.5 mM calcium (alone or as Aquamin) demonstrated a larger number of desmosomes between cells. Individual desmosomes were wider and more electron dense. Well-organized intermediate filaments connected to the desmosomes were apparent in places. Findings were similar with all three subjects. Efforts to quantify desmosomal number (Fig 6d) confirmed the higher number with intervention as compared to control.

Tight junctional complexes (black arrows) were also evident by electron microscopy (Fig 6a–6c). These were present at the luminal surface between virtually every cell, regardless of whether the colonoids were cultured under control conditions or exposed to either intervention. This is consistent with the proteomic and immunohistological findings (modest up-regulation of tight junctional proteins), and is in sharp contrast to what was observed previously in adenoma colonoids. In the adenoma colonoids, there were few detectable tight junctions under low-calcium conditions. However, when exposed to higher calcium concentrations, tight junctions were apparent [33].

## Discussion

In the present study, colonoid cultures obtained from histologically-normal colon tissue were examined under low-calcium (0.25 mM) conditions and compared to colonoids treated with a range of calcium concentrations (1.5–3.0 mM) from calcium alone or Aquamin. These interventions were recently shown to induce differentiation and suppress proliferation in adenoma colonoids [33]. Compared to what was observed with adenoma colonoids, where a high proliferation index and minimal differentiation were observed in the absence of intervention (calcium alone or Aquamin), normal tissue colonoids demonstrated a highly-differentiated phenotype in the absence of intervention. Specifically, under both low-calcium conditions and in response to intervention, colonoids appeared either as thin-walled, translucent “cystic” structures or differentiated crypts. The cystic structures reflect colonoids enriched in stem cells and are due to the high level of Wnt-pathway ligands in the culture medium (L-WRN) used to maintain normal tissue colonoids [32–36]. What accounts for the low sensitivity of the adenoma-derived colonoids to low ambient calcium is unclear. While higher calcium (1.5–3.0 mM) from either intervention induced only modest additional differentiation in normal colonoids, adenoma colonoids were highly differentiated in response to these interventions. The potential of epithelial cells in the adenomas to undergo differentiation in response to higher calcium concentrations suggests that even after the premalignant tumors have reached the “large adenoma” size, there is still potential for calcium-supplementation to have a pro-differentiating effect. This is of interest because it has been shown in past studies [54,55] with human colon adenomas that a histologically-differentiated presentation is a favorable prognostic factor.

Even after (premalignant) colon adenomas have progressed to the invasive cancer (malignant) stage, a differentiated appearance still has favorable prognostic significance [56–58]. Why this should be so, is not fully understood. The better prognosis may reflect reduced proliferation associated with the more differentiated phenotype (as shown above and in our recent study [33]). Alternatively, improved prognosis may reflect a better response to chemotherapy. A recent analysis of several past studies showed, for example, that adjuvant 5-fluorouracil (5-FU) was beneficial in localized rectal cancer and that the addition of oxaliplatin marginally improved outcome over that seen with 5-FU alone [59]. This is of interest in the present context because thymidylate synthase, the target enzyme of 5-FU, is a calcium-sensitive moiety. Our own past studies showed that enzyme down-regulation with calcium treatment improved the response of colon carcinoma cells to 5-FU [60]. Additionally, it has been shown that oxaliplatin down-regulates survivin [61,62], a protein that protects tumor cells from apoptosis. Like thymidylate synthase, survivin is strongly sensitive to calcium [60]. Glucose-6 phosphate dehydrogenase provides another potential target for adjuvant intervention [63]. This protein is up-regulated in many human tumors and is sensitive to oncosuppressors such as p53 [64]. Our own recent study with human adenoma colonoids demonstrated up-regulation of suppressor pathways with calcium [33].

While such studies suggest benefit for calcium even after progression of colon adenomas to the fully malignant state, chemoprevention with non-toxic interventions may be a more viable option [65–68]. A long term goal of our work has been to determine if the inclusion of additional trace elements along with calcium can improve on the efficacy of calcium alone as a colon cancer chemopreventive agent. Utilizing a natural product, i.e.,—Aquamin—that contains a high concentration of magnesium as well as measurable levels of 72 additional trace elements in addition to calcium, we have shown improved colon polyp prevention in long-term animal studies [27,28] compared to calcium alone. Better epithelial growth suppression in cell culture [25,26] has also been observed. Consistent with these findings, Aquamin (at a level providing only 0.15 mM calcium) suppressed growth and induced features of differentiation in adenoma colonoids not seen with calcium alone at a comparable level [33]. In contrast to these past observations with abnormal epithelium, here we show in colonoids derived from histologically-normal tissue that extensive differentiation occurred in the absence of intervention, and that treatment with either calcium alone or Aquamin had only a modest additional effect. The overall lack of widespread differences between the two interventions in the normal colonic cultures makes it unlikely that Aquamin will have a negative impact on the normal colonic mucosa when used as a chemopreventive agent.

Although intervention with either calcium alone or Aquamin produced an only modest change in the gross and histological appearance of normal tissue-derived colonoids, there was a strong induction of several proteins that contribute to cellular adhesive functions, barrier formation and tissue integrity. Among these were laminin subunits, nidogen, a fibronectin domain protein, Integrin alpha V and beta 5 subunits and a number of CEACAMS (cell-matrix adhesion), cadherin family members (adherens junctions), claudins (tight junctions) and desmosomal proteins including desmoglein-2, desmocollin-2, desmoplakin and PERP. At the ultrastructural level, a large number of actual desmosomes along the lateral cell-cell boundary could be seen in colonoids exposed to the interventions. These structures were sparse in colonoids cultured under low-calcium conditions.

**Fig 7:**
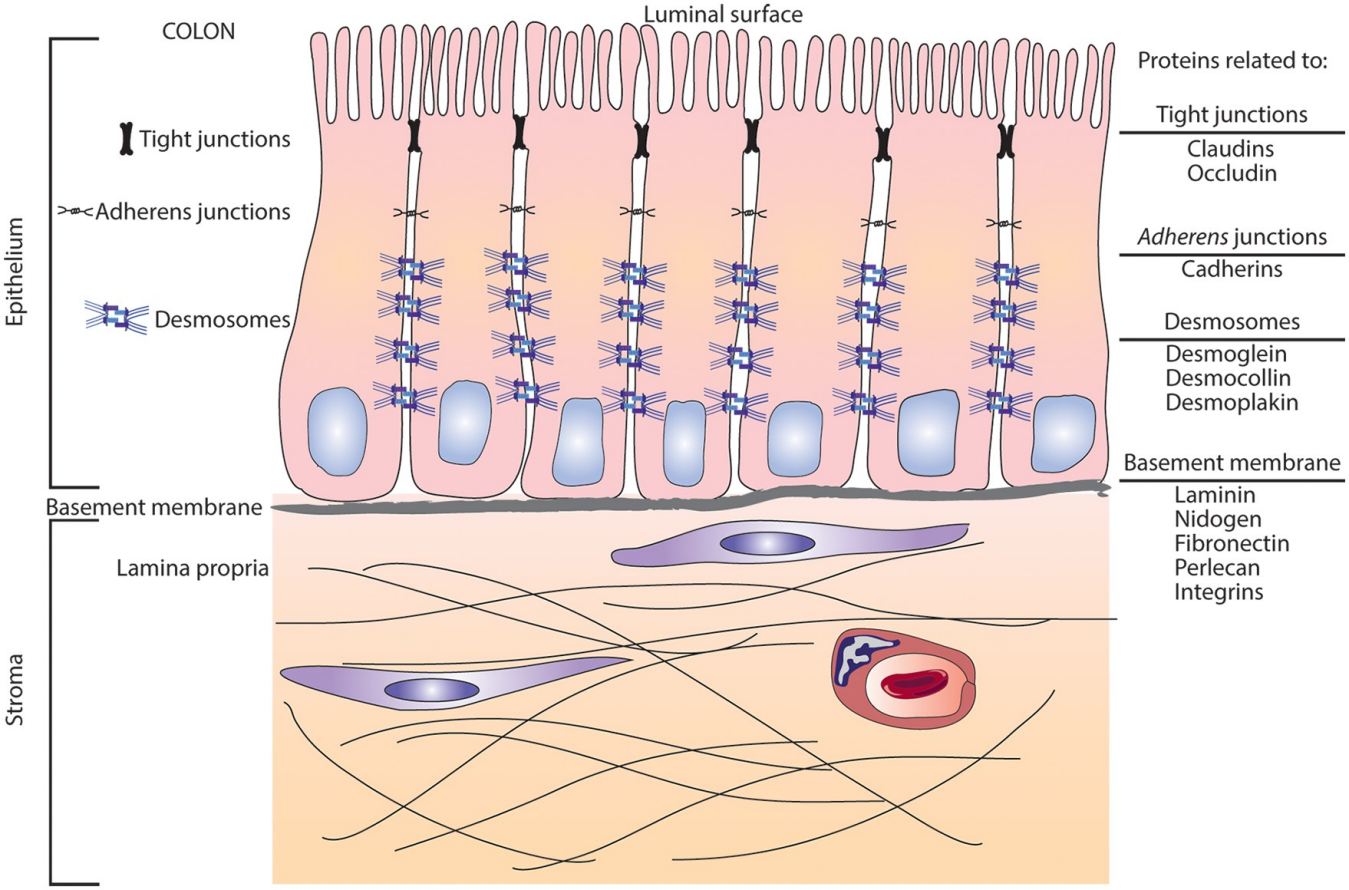
Schematic representation of structures involved in formation of the barrier in the colonic mucosa. An infographic with a display of structures and proteins involved in the formation and enhancement of the cell barrier as present in the human gut.

The implication of these findings goes beyond the normal colonocyte—tumor cell transformation process. All of these cell-cell and cell-matrix adhesion proteins are needed for barrier formation and tissue integrity. Tight junctions and adherens junctions form the barrier at the luminal side of the colonic epithelium, while the cell-matrix adhesion molecules form the barrier at the basement membrane between the epithelium and interstitium. Barriers on both sides depend on cell-cell cohesive strength provided by desmosomes [69] as depicted schematically in Fig 7. Inadequacies in any of these barrier-related proteins compromise tissue integrity and likely contribute to interstitial infiltration by bacteria, bacterial toxins and food allergens; all of which are capable of provoking an inflammatory response [70]. In addition to a direct role in barrier formation / tissue integrity, a recent study has indicated that certain of these proteins (specifically, desmoglein 2) participate in signaling pathways that regulate growth and differentiation [71]. Thus, impairment of these proteins may have a role in the pathogenesis of inflammatory bowel diseases [72]. Not surprisingly, proteins (Intestinal alkaline phosphatase and 15-hydroxyprostaglandin dehydrogenase) known to have an anti-inflammatory role in the colon [43–45] were concomitantly up-regulated with the barrier proteins.

That all of these critical structures are up-regulated with calcium supplementation (even though morphological features associated with the differentiated phenotype were seen under low-calcium control conditions) attests to the importance of having an adequate calcium-intake to gain maximal benefits. Unfortunately, the Western-style diet is deficient in the level of calcium provided to most individuals. This has been well-documented in studies from North America, Europe and Australia [73,74]. Additionally, it has been shown that even a substantial percentage of individuals on a mostly plant-based diet do not achieve an adequate calcium intake [75]. Calcium is not the only mineral in which there is a wide-spread deficiency. Approximately 50% of the US population does not meet the US recommended daily allowance for dietary intake of magnesium [76,77]. While many of the other minerals represented in Aquamin are not routinely tested for and have no daily recommended intake levels, it may be assumed that individuals who do not achieve adequate calcium and magnesium levels would also be deficient in other elements that are nutritionally associated with these elements. Whether or not routine dietary mineral supplementation would be an effective way to mitigate some of the age-related chronic diseases remains to be seen in controlled clinical trials. Such studies are in progress in our laboratory.

There were some limitations to the study. The small sample size (n = 5) was one. In spite of this, we were able to demonstrate several important statistically significant differences among treatment groups. This reflects the consistency among specimens from different subjects and is in contrast to what we found earlier in our analysis of adenoma colonoid cultures [33]. Another limitation was the lack of functional assays to accompany the immunohistochemistry and proteomic findings. Studies to assess functional changes in colonoid behavior—i.e., transepithelial resistance (TER) and tissue integrity assays—will be conducted to validate these findings. Finally, and perhaps most importantly, this was an *ex vivo* system. Whether similar findings to those described here will be seen following *in vivo* intervention is not known. We will be able to address this issue eventually, however, because we are currently involved in a 90-day interventional trial comparing calcium alone to Aquamin for effects on the same colonic endpoints as studied here in culture (clinicaltrials.gov: NCT02647671).

## Conclusions

In summary, the studies described here demonstrate that colonoids obtained from histologically-normal colon tissue express gross and histological features of differentiation in the presence of a low ambient level of extracellular calcium. In this respect, the normal tissue colonoids are different from the previously-studied adenoma colonoids [33], which demonstrated little evidence of differentiation (and a high proliferation index) under low-calcium conditions, but differentiated in response to calcium supplementation. Taken together, these findings suggest that reduced sensitivity to low ambient calcium is an inherent feature of the transformation process in the colonic epithelium. In addition, the present findings show that even in colonoids that are already differentiated based on morphological criteria, intervention with calcium up-regulates proteins that contribute to adhesive function, barrier formation and tissue integrity. To the extent that dietary mineral supplementation can improve barrier formation and tissue integrity in the gastrointestinal tract, it should help reduce chronic inflammation and, ultimately, mitigate some of the age-related conditions that result from chronic inflammation.

## Supporting information

S1 Fig Colonoid appearance in culture: Phase-contrast microscopy (higher concentrations– 2.1 and 3.0 mM). (TIF)

S2 Fig Immunostained sections of colonoids (higher concentrations—2.1 and 3.0 mM). (TIF)

S3 Fig Protein-Protein interactions—String-database. (TIF)

S1 Table Characteristics of subjects providing tissue. (DOCX)

S2 Table Antibody characteristics. (DOCX)

S3 Table Pathways associated with up- and down-regulated proteins (unbiased proteomic analysis). (DOCX)

S4 Table Additional differentiation-related proteins (based on proteomic analysis). (DOCX)
