# Supplementary figures and images for: Calcium-induced differentiation in normal human colonoid cultures: Cell-cell / cell-matrix adhesion, barrier formation and tissue integrity
Source: PLoS One. 2019 Apr 17;14(4):e0215122. doi: 10.1371/journal.pone.0215122 (PMC6469792; doi:10.1371/journal.pone.0215122)

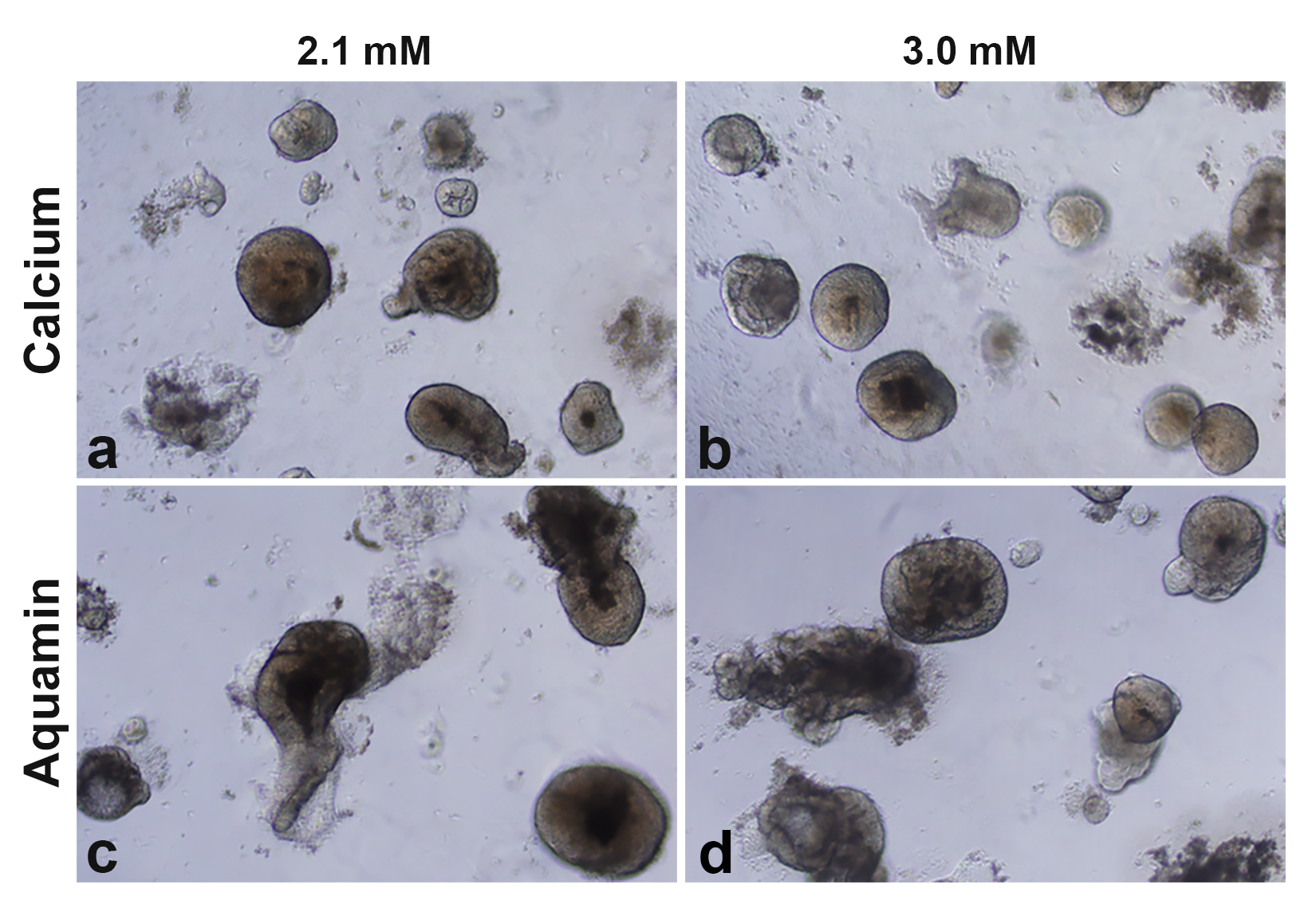

Supplement: S1 Fig — (TIF) [file pone.0215122.s001.tif]

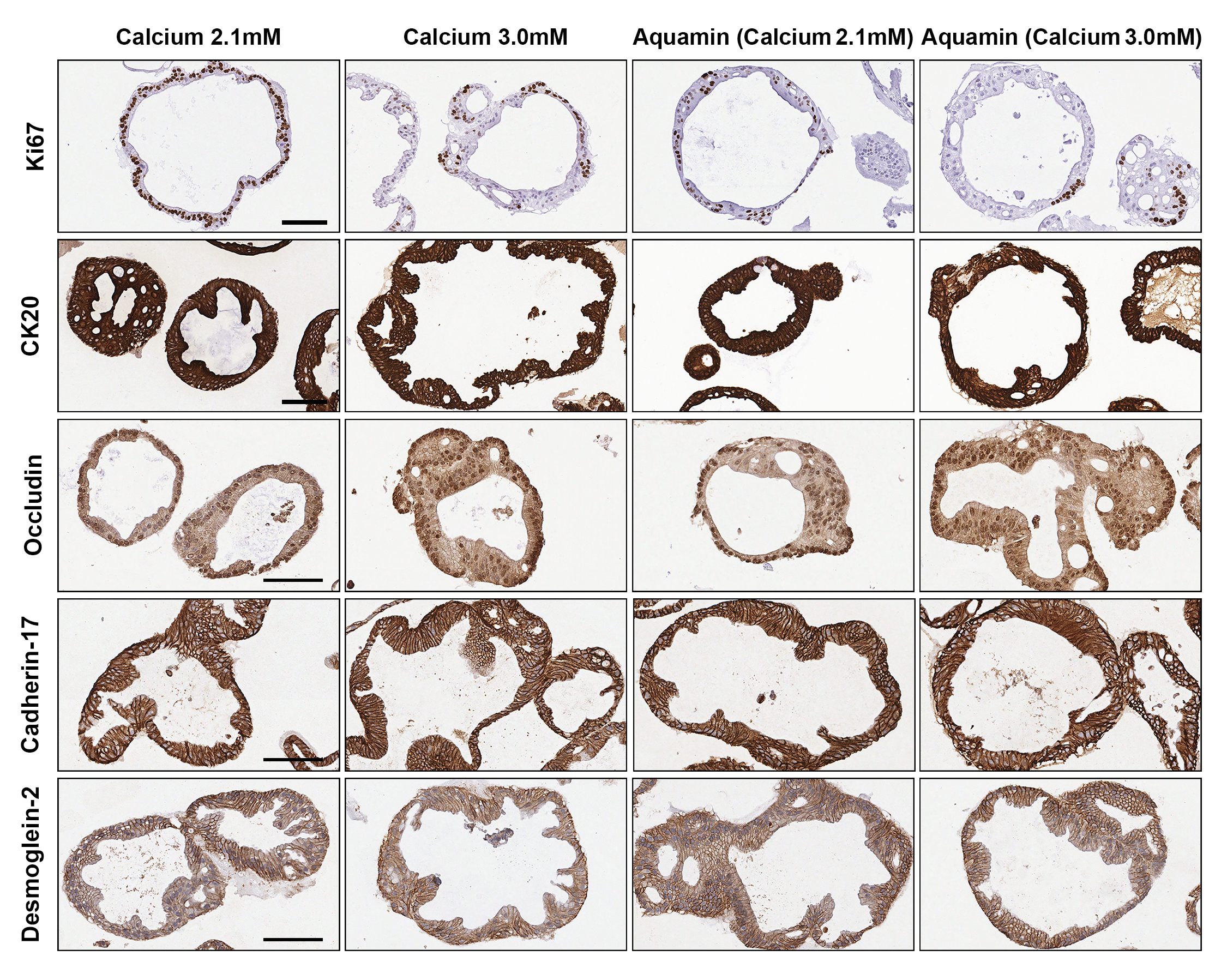

Supplement: S2 Fig — (TIF) [file pone.0215122.s002.tif]

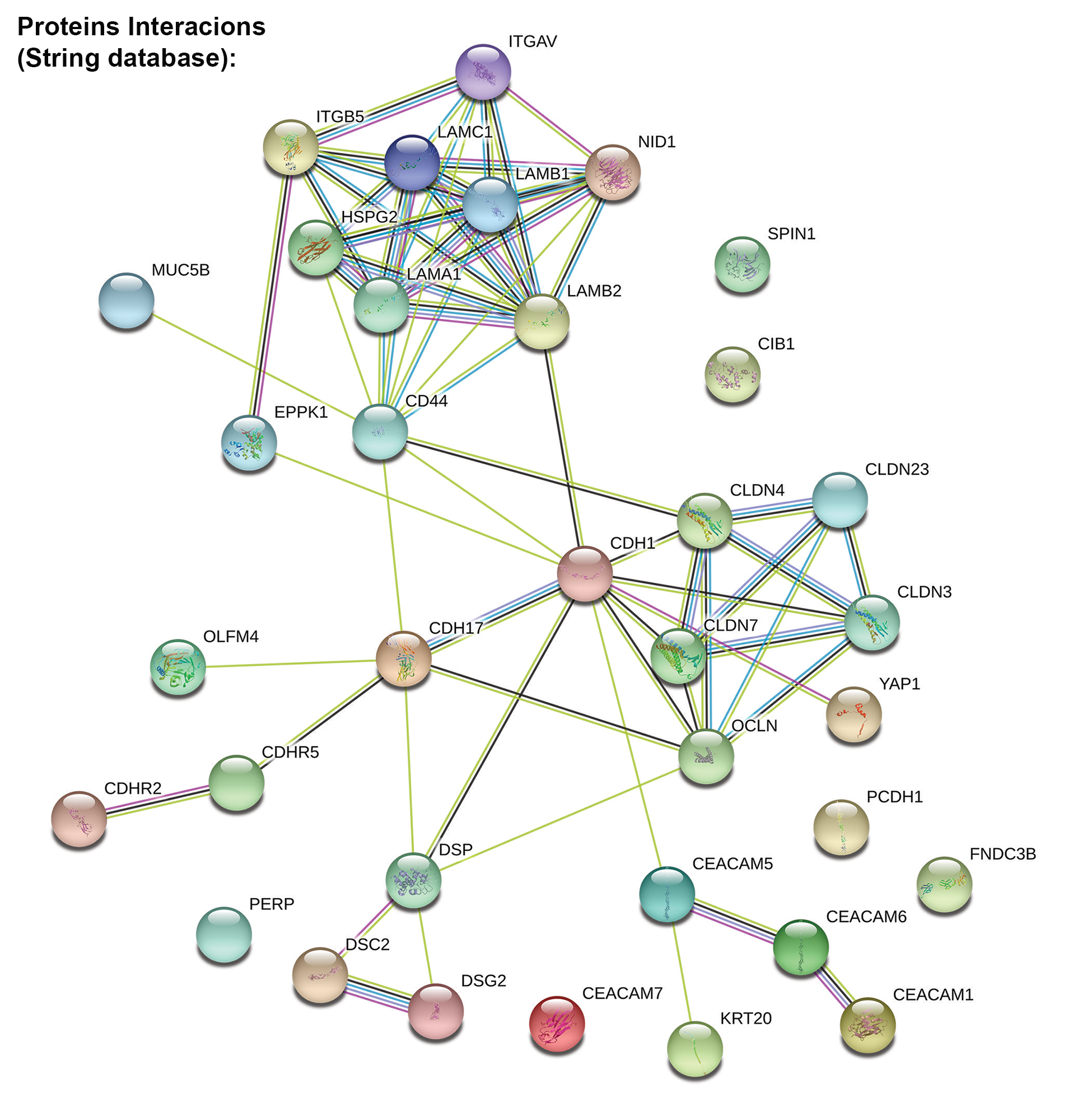

Supplement: S3 Fig — (TIF) [file pone.0215122.s003.tif]
