# Supplementary material for: Calcium-induced differentiation in normal human colonoid cultures: Cell-cell / cell-matrix adhesion, barrier formation and tissue integrity
Source: PLoS One. 2019 Apr 17;14(4):e0215122. doi: 10.1371/journal.pone.0215122 (PMC6469792; doi:10.1371/journal.pone.0215122)
Supplement: S1 Table — (DOCX) [file pone.0215122.s004.docx]

| **S1 Table. Human Colon Samples (Used in this study).** | | | | | | | |
| --- | --- | --- | --- | --- | --- | --- | --- |
| **Subject ID** | **Age** (Years) **/Sex** | **Location** | **Eligibility criteria** |  |  |  |  |
| 90 | 31/F | Sigmoid colon | H/o colon polyp, Family h/o CRC (First degree relative) | | | | |
| 104 | 58/F | Sigmoid colon | H/o colon polyp, Family h/o CRC (First degree relative) | | | | |
| 106 | 50/M | Sigmoid colon | H/o colon polyp | | | | |
| 108 | 61/M | Sigmoid colon | H/o colon polyp, Family h/o CRC (First degree relative) | | | | |
| 111 | 20/M | Sigmoid colon | Family h/o CRC (First degree relative) | | | | |
| ___________________________________________________________________________________________ | | | | | | | |

H/o = History of, CRC = Colorectal Cancer.
