# Supplementary material for: Calcium-induced differentiation in normal human colonoid cultures: Cell-cell / cell-matrix adhesion, barrier formation and tissue integrity
Source: PLoS One. 2019 Apr 17;14(4):e0215122. doi: 10.1371/journal.pone.0215122 (PMC6469792; doi:10.1371/journal.pone.0215122)
Supplement: S2 Table — (DOCX) [file pone.0215122.s005.docx]

| **S2 Table. Antibodies used in the study (for IHC assay)** | | | | | |
| --- | --- | --- | --- | --- | --- |
| **Antibody** | **Vendor** | **Catalog #** | **Dilution** | **Incubation Time** | **Retrieval Method** |
| Rb Ki67 MaB clone SP6 | Cell Marque | 475 R-16 | 1:250 | 30 min | FLEX TRS High pH (9.01), 20 min |
| Ms CK20 MaB clone Ks20.8 | Dako | M7019 | 1:100 | 60 min | FLEX TRS High pH (9.01), 20 min |
| Rb LI Cadherin (CDH17) MaB  clone EPR3996 | AbCam | Ab109190 | 1:250 | 60 min | ^a^HIER pH 9.0 |
| Rb Desmoglein 2 PaB | Sigma | HPA004896 | 1:200 | 30 min | ^b^HIER pH 6.0 |
| Rb Occludin PaB | Invitrogen | 71-1500 | 1:250 | 60 min | pFLEX TRS Low pH (6.10), 20 min |
| _______________________________________________________________________________________________________ | | | | | |
| ^a^HIER pH 9: Heat induced epitope retrieval 10 mM Tris HCl/1 mM EDTA buffer pH9  ^b^HIER pH 6: Heat induced epitope retrieval Citrate buffer pH6 | | | | | |
|  | | | | | |
|  | |  |  |  |  |
