# Supplementary material for: Calcium-induced differentiation in normal human colonoid cultures: Cell-cell / cell-matrix adhesion, barrier formation and tissue integrity
Source: PLoS One. 2019 Apr 17;14(4):e0215122. doi: 10.1371/journal.pone.0215122 (PMC6469792; doi:10.1371/journal.pone.0215122)
Supplement: S3 Table — (DOCX) [file pone.0215122.s006.docx]

| **S3A Table. Pathways associated with Up-regulated proteins (Reactome v66)** |  |
| --- | --- |
|  |  |
| **Pathway name** | **Entities pValue** |
| Formation of the cornified envelope | 0.0000001 |
| Keratinization | 0.000002 |
| Post-translational modification: synthesis of GPI-anchored proteins | 0.00001 |
| SLC transporter disorders | 0.00004 |
| Multifunctional anion exchangers | 0.0003 |
| Neutrophil degranulation | 0.0004 |
| Type I hemidesmosome assembly | 0.0005 |
| SLC-mediated transmembrane transport | 0.001 |
| Disorders of transmembrane transporters | 0.001 |
| Transport of small molecules | 0.001 |
| Cell junction organization | 0.002 |
| Metal ion SLC transporters | 0.003 |
| Defective SLC39A4 causes acrodermatitis enteropathica, zinc-deficiency type (AEZ) | 0.003 |
| Defective SLC11A2 causes hypochromic microcytic anemia, with iron overload 1 (AHMIO1) | 0.003 |
| Defective SLC26A2 causes chondrodysplasias | 0.003 |
| Defective SLC26A3 causes congenital secretory chloride diarrhea 1 (DIAR1) | 0.003 |
| Transport of inorganic cations/anions and amino acids/oligopeptides | 0.004 |
| Cell-Cell communication | 0.006 |
| Proton/oligopeptide cotransporters | 0.011 |
| Biosynthesis of D-series resolvins | 0.011 |
| Alternative complement activation | 0.014 |
| Biosynthesis of E-series 18(S)-resolvins | 0.014 |
| Mtb iron assimilation by chelation | 0.017 |
| Transport and synthesis of PAPS | 0.017 |
| Metal sequestration by antimicrobial proteins | 0.017 |
| Synthesis of Lipoxins (LX) | 0.017 |
| Biosynthesis of EPA-derived SPMs | 0.017 |
| Activation of C3 and C5 | 0.020 |
| Erythrocytes take up oxygen and release carbon dioxide | 0.023 |
| Synthesis of Ketone Bodies | 0.023 |
| HDL assembly | 0.023 |
| Transport of bile salts and organic acids, metal ions and amine compounds | 0.026 |
| Developmental Biology | 0.028 |
| Zinc influx into cells by the SLC39 gene family | 0.028 |
| Ketone body metabolism | 0.028 |
| Vitamin D (calciferol) metabolism | 0.031 |
| Apoptotic cleavage of cell adhesion proteins | 0.031 |
| Reversible hydration of carbon dioxide | 0.034 |
| O2/CO2 exchange in erythrocytes | 0.034 |
| Erythrocytes take up carbon dioxide and release oxygen | 0.034 |
| Post-translational protein phosphorylation | 0.038 |
| Synthesis of Prostaglandins (PG) and Thromboxanes (TX) | 0.042 |
| Innate Immune System | 0.048 |
| Vitamin B5 (pantothenate) metabolism | 0.048 |
| Zinc transporters | 0.048 |
| Metabolism of Angiotensinogen to Angiotensins | 0.048 |
| Biosynthesis of DHA-derived SPMs | 0.048 |
| Regulation of Insulin-like Growth Factor transport and uptake by Insulin-like Growth Factor Binding Proteins | 0.050 |
| ______________________________________________________________________________________________________ | |

These pathways are based on the up-regulated proteins (1.8 fold-change) presented in Table 1.

| **S3B Table. Pathways associated with Down-regulated proteins (Reactome v66)** |  |
| --- | --- |
|  |  |
| **Pathway name** | **Entities pValue** |
| TP53 Regulates Transcription of Cell Cycle Genes | 0.0005 |
| Chromosome Maintenance | 0.0018 |
| Nonsense-Mediated Decay (NMD) | 0.003 |
| Nonsense Mediated Decay (NMD) enhanced by the Exon Junction Complex (EJC) | 0.003 |
| Infectious disease | 0.003 |
| TFAP2A acts as a transcriptional repressor during retinoic acid induced cell differentiation | 0.004 |
| Sensing of DNA Double Strand Breaks | 0.004 |
| DNA Double-Strand Break Repair | 0.005 |
| Influenza Infection | 0.006 |
| Regulation of expression of SLITs and ROBOs | 0.006 |
| SUMO E3 ligases SUMOylate target proteins | 0.006 |
| SUMOylation | 0.007 |
| Removal of the Flap Intermediate from the C-strand | 0.007 |
| Processive synthesis on the C-strand of the telomere | 0.008 |
| Purine ribonucleoside monophosphate biosynthesis | 0.009 |
| Signaling by ROBO receptors | 0.010 |
| Removal of the Flap Intermediate | 0.010 |
| Polymerase switching | 0.010 |
| Leading Strand Synthesis | 0.010 |
| Polymerase switching on the C-strand of the telomere | 0.010 |
| Mismatch repair (MMR) directed by MSH2:MSH3 (MutSbeta) | 0.010 |
| Mismatch repair (MMR) directed by MSH2:MSH6 (MutSalpha) | 0.010 |
| Processive synthesis on the lagging strand | 0.011 |
| Mismatch Repair | 0.011 |
| Nucleobase biosynthesis | 0.011 |
| Translesion synthesis by REV1 | 0.011 |
| Translesion synthesis by POLI | 0.012 |
| Translesion synthesis by POLK | 0.012 |
| TP53 Regulates Transcription of Genes Involved in G2 Cell Cycle Arrest | 0.013 |
| Gene expression (Transcription) | 0.013 |
| Translesion Synthesis by POLH | 0.014 |
| Transcription of E2F targets under negative control by DREAM complex | 0.014 |
| Lagging Strand Synthesis | 0.014 |
| PCNA-Dependent Long Patch Base Excision Repair | 0.015 |
| TP53 regulates transcription of additional cell cycle genes | 0.015 |
| Telomere C-strand (Lagging Strand) Synthesis | 0.017 |
| Gap-filling DNA repair synthesis and ligation in GG-NER | 0.018 |
| Resolution of AP sites via the multiple-nucleotide patch replacement pathway | 0.018 |
| DNA Repair | 0.018 |
| G0 and Early G1 | 0.019 |
| Activation of E2F1 target genes at G1/S | 0.020 |
| G1/S-Specific Transcription | 0.020 |
| Extension of Telomeres | 0.021 |
| Recognition of DNA damage by PCNA-containing replication complex | 0.022 |
| RNA Polymerase I Transcription Termination | 0.022 |
| Termination of translesion DNA synthesis | 0.023 |
| DNA strand elongation | 0.023 |
| Nuclear import of Rev protein | 0.026 |
| Transcriptional regulation by the AP-2 (TFAP2) family of transcription factors | 0.026 |
| Transcriptional Regulation by TP53 | 0.027 |
| Translesion synthesis by Y family DNA polymerases bypasses lesions on DNA template | 0.028 |
| Resolution of Abasic Sites (AP sites) | 0.028 |
| Base Excision Repair | 0.028 |
| Interactions of Rev with host cellular proteins | 0.028 |
| Dual Incision in GG-NER | 0.029 |
| NS1 Mediated Effects on Host Pathways | 0.031 |
| SUMOylation of transcription cofactors | 0.031 |
| Host Interactions with Influenza Factors | 0.034 |
| SUMOylation of DNA replication proteins | 0.034 |
| DNA Damage Bypass | 0.035 |
| Deposition of new CENPA-containing nucleosomes at the centromere | 0.038 |
| Nucleosome assembly | 0.038 |
| mRNA 3'-end processing | 0.040 |
| E3 ubiquitin ligases ubiquitinate target proteins | 0.042 |
| DNA Double Strand Break Response | 0.042 |
| Disease | 0.043 |
| Telomere Maintenance | 0.045 |
| Gap-filling DNA repair synthesis and ligation in TC-NER | 0.045 |
| Dual incision in TC-NER | 0.046 |
| RNA Polymerase II Transcription Termination | 0.047 |
| Cleavage of Growing Transcript in the Termination Region | 0.047 |
| HDR through Homologous Recombination (HRR) | 0.047 |
| __________________________________________________________________________________________________________________________ | |

These pathways are based on the down-regulated proteins (1.8 fold-change) presented in Table 2.
