# Supplementary material for: Calcium-induced differentiation in normal human colonoid cultures: Cell-cell / cell-matrix adhesion, barrier formation and tissue integrity
Source: PLoS One. 2019 Apr 17;14(4):e0215122. doi: 10.1371/journal.pone.0215122 (PMC6469792; doi:10.1371/journal.pone.0215122)
Supplement: S4 Table — (DOCX) [file pone.0215122.s007.docx]

**S4 Table. Additional differentiation-related (Keratins and Integrins) proteins**

|  | **Calcium** | **Calcium** | **Calcium** | **Aquamin** | **Aquamin** | **Aquamin** |
| --- | --- | --- | --- | --- | --- | --- |
| **Proteins** | **1.5mM** | **2.1mM** | **3.0mM** | **(1.5mM** | **(2.1mM** | **(3.0mM** |
|  |  |  |  | **Calcium)** | **Calcium)** | **Calcium)** |
| **Keratin family proteins** |  |  |  |  |  |  |
| ^#^Keratin, type I cytoskeletal 9 | 1.07±0.47 | 1.79±1.73 | *0.59±0.13 | 3.66±4.03 | 9.96±15.39 | 0.98±0.34 |
| Keratin, type I cytoskeletal 10 | 0.88±0.30 | 1.11±0.57 | 0.87±0.48 | 1.75±1.74 | 2.91±1.90 | 0.86±0.35 |
| Keratin, type I cytoskeletal 13 | 1.07±0.70 | 0.98±0.29 | 0.83±0.36 | 0.89±0.39 | 2.25±3.16 | 1.09±0.78 |
| ^#^Keratin, type I cytoskeletal 14 | 0.84±0.25 | 3.33±3.45 | 1.24±0.71 | 1.88±1.89 | 4.36±2.68 | 0.99±0.14 |
| ^#^Keratin, type I cytoskeletal 16 | 0.98±0.14 | 3.88±4.88 | 1.00±0.14 | 5.59±8.08 | 8.11±7.19 | 0.92±0.22 |
| Keratin, type I cytoskeletal 18 | 0.91±0.20 | 0.91±0.22 | 1.01±0.20 | 0.83±0.11 | 0.97±0.29 | 0.93±0.13 |
| Keratin, type I cytoskeletal 19 | 1.20±0.32 | 1.19±0.28 | 1.43±0.15 | 1.09±0.18 | 1.19±0.26 | 1.18±0.09 |
| ^#^Keratin, type I cytoskeletal 20 | 1.42±0.33 | *1.46±0.21 | *1.62±0.06 | 1.42±0.33 | *1.47±0.24 | 1.35±0.15 |
| Keratin, type I cytoskeletal 17 | 0.76±0.16 | 1.59±0.94 | 1.20±0.40 | 1.27±0.41 | 1.59±0.49 | 0.96±0.06 |
| ^#^Keratin, type II cytoskeletal 1 | 0.94±0.30 | 1.58±1.41 | 0.73±0.34 | 2.79±3.22 | 7.12±8.56 | 0.95±0.12 |
| Keratin, type II cytoskeletal 2 | 1.03±0.22 | 1.22±0.36 | 1.05±0.38 | 2.31±2.57 | 2.15±0.89 | 0.99±0.47 |
| ^#^Keratin, type II cytoskeletal 5 | 1.14±0.34 | 1.47±0.60 | 1.08±0.15 | 2.83±3.09 | 4.50±3.04 | 0.98±0.02 |
| ^#^Keratin, type II cytoskeletal 6A | 0.92±0.16 | 1.80±1.59 | 0.98±0.07 | 3.95±5.37 | 4.95±5.38 | 1.07±0.25 |
| Keratin, type II cytoskeletal 6B | *0.59±0.13 | 0.80±0.16 | 0.45±0.27 | 4.03±5.78 | 3.56±4.71 | 0.80±0.22 |
| Keratin, type II cytoskeletal 7 | 0.93±0.06 | 0.93±0.07 | 0.99±0.02 | 0.90±0.11 | 0.95±0.22 | 0.94±0.03 |
| Keratin, type II cytoskeletal 8 | 1.22±0.33 | 1.22±0.26 | *1.44±0.08 | *1.14±0.07 | 1.26±0.27 | *1.25±0.00 |
| **Cell-matrix adhesion proteins** |  |  |  |  |  |  |
| Integrin alpha-2 | 0.91±0.12 | 0.85±0.06 | 0.81±0.01 | 0.92±0.18 | 0.81±0.01 | 0.84±0.01 |
| Integrin alpha-3 | 0.91±0.07 | 0.93±0.04 | 0.97±0.03 | 0.90±0.06 | 0.82±0.04 | 0.88±0.04 |
| Integrin alpha-6 | 0.93±0.05 | 0.93±0.02 | 0.89±0.06 | 0.94±0.09 | 0.88±0.09 | 0.97±0.08 |
| ^#^Integrin alpha-V | 1.22±0.15 | *1.23±0.14 | *1.21±0.07 | *1.19±0.10 | 1.19±0.13 | 1.19±0.07 |
| Integrin beta-1 | 0.97±0.08 | 0.96±0.06 | 0.98±0.03 | 0.93±0.05 | 0.88±0.04 | 0.96±0.02 |
| Integrin beta-4 | 0.96±0.13 | 0.93±0.13 | 0.91±0.12 | 0.92±0.05 | 0.89±0.07 | 0.93±0.07 |
| ^#^Integrin beta-5 | 1.27±0.28 | 1.17±0.11 | *1.07±0.02 | 1.13±0.08 | 1.23±0.18 | *1.11±0.01 |
| _____________________________________________________________________________________ | | | | | | |

Values represent average fold-change across specimens from three different subjects. Specimens from each subject were assessed separately and fold-change with each intervention compared to control (0.25 mM calcium). Then values from each intervention for all three subjects were averaged to generate the average fold-change values shown. Asterisks indicate statistical significance at p<0.05 based on student t-test with correction for multiple comparisons. ^#^ is placed to represent proteins which have included in the Tables 1 and 3.
